# The Structure of Human IAPP Fibrils Reflects Membrane and pH Conditions

**DOI:** 10.1021/jacs.5c06971

**Published:** 2025-08-01

**Authors:** Venus Singh Mithu, Karin Giller, Evgeny Nimerovsky, Kerstin Overkamp, Loren B. Andreas, Stefan Becker, Christian Griesinger

**Affiliations:** Department of NMR-based Structural Biology, 28282 Max Planck Institute of Multidisciplinary Sciences, Göttingen 37077, Germany

## Abstract

Physiologically relevant in vitro models of amyloid aggregation are essential for linking structural insights to disease pathology. In type 2 diabetes, aggregation of human islet amyloid polypeptide (hIAPP) into fibrils is a hallmark of β-cell dysfunction, yet structural data on ex vivo hIAPP fibrils remain unavailable. Most models use solution-grown fibrils, overlooking membrane interactions and native pH, which underscores the need for more realistic in vitro models. Here, we use solid-state NMR spectroscopy to determine the structure of phospholipid membrane-mediated hIAPP fibrils formed under extracellular (pH 7.4) conditions. These fibrils are homogeneous and adopt an L-shaped protofilament architecture with an extended N-terminal β-strand—a region often unresolved in cryo-EM. The fibril core (N14–L27) adopts the CF1 fold, a conserved β-arch also seen in nonlipidic fibrils, suggesting its relevance in disease. In contrast, fibrils formed at intracellular pH (5.3) are structurally heterogeneous and show distinct structural differences in the C-terminus. hIAPP must exhibit substantial structural plasticity in the membrane environment, transitioning from helical monomers to β-hairpin oligomers and ultimately to β-arch-rich fibrils—transitions that may introduce energy barriers stabilizing toxic intermediates. Our findings provide the first high-resolution structure of membrane-mediated hIAPP fibrils highlighting the need to model aggregation under physiologically relevant conditions.

## Graphical abstract

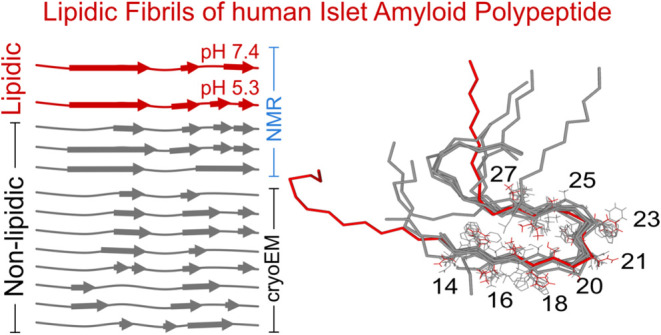

## Introduction

hIAPP, also known as amylin, is a 37-residue peptide hormone. In pancreatic β-cells, it is stored alongside insulin in acidic secretory granules (∼pH 5.3) and cosecreted into the neutral extracellular space (∼pH 7.4).^1^ It plays a pivotal role in glucose homeostasis by slowing gastric emptying, modulating gastric motility, and suppressing postprandial glucagon release.^2^ However, under pathological conditions, hIAPP aggregates into insoluble amyloid fibrils, which are prominent in the pancreatic islets of individuals with type 2 diabetes mellitus (T2DM).^3^ This aggregation is a major contributor to β-cell dysfunction and death, which exacerbates disease progression.^4^

Despite its role in disease, no structural details of ex vivo hIAPP fibrils are available. While high-resolution structures of patient-derived fibrils in amyloidogenic diseases such as Alzheimer’s (AD) and Parkinson’s (PD) are available,^5^ similar insights for T2DM remain elusive. Since the early 2000s, several atomic-level structures of in vitro hIAPP fibrils—particularly using solid-state nuclear magnetic resonance (ssNMR) spectroscopy—have been proposed.^6–8^ More recently, advances in cryo-electron microscopy (cryo-EM) have led to seven high-resolution structures of in vitro hIAPP fibrils in the past five years.^9–15^ However, these studies examined fibrils grown from solution under conditions optimized for structural quality rather than physiological relevance.^9^ This underscores the need for improved in vitro models to better understand fibril formation and support the development of therapeutic strategies.

Amyloid aggregation is a multistep process involving nucleation, elongation, and fibril maturation.^16^ Cellular membranes are central to hIAPP aggregation,^17^ acting both as targets of toxic oligomeric intermediates^18,19^ and nucleation sites for fibril formation.^20–22^ While some studies link membrane toxicity to pore formation,^19,23^ others attribute it to fibril-growth-induced damage.^24^ Despite the unclear mechanism,^25^ in vitro studies demonstrate that phospholipid membranes significantly influence hIAPP aggregation kinetics and morphology, with effects varying based on membrane composition and buffer conditions.^17,22,26,25^ Among these factors, pH plays a crucial role, shaping hIAPP’s aggregation potential and membrane-damaging capacity, while fueling the debate on whether fibrils form intracellularly or after secretion.^28,29^ Physiologically relevant pH and cell-membrane mimics are critical factors yet often overlooked in structural studies.^28^ Except for a single solution NMR study analyzing the structure of a phospholipid nanodisc-bound hIAPP oligomer,^30^ high-resolution structures of lipidic hIAPP aggregates remain unavailable. Our study addresses this gap by incorporating phospholipid membranes at pH 5.3 and 7.4, providing a more accurate in vitro model for structural insights and therapeutic development.

Lipidic fibrils appear densely clumped, with interspersed lipid membranes seemingly acting as a “glue” (Figure 1). These tightly packed fibril clusters, spanning several micrometers in one dimension, stand in stark contrast to the well-dispersed fibrils observed without lipid membranes (Figure S1). A similar dispersed morphology has been observed in fibrils seeded from patient-derived material in the absence of lipids, in contrast to the distinct, clustered architecture of lipid-associated fibrils seen here.^13^ This clumping disrupts the even distribution of fibrils on cryo-EM grids, which is critical for the stability of thin vitreous ice films, posing significant challenges for structural characterization using cryo-EM.^9^ In this study, we used recombinantly prepared hIAPP with a C-terminal amide and C2–C7 disulfide bond, applying magic-angle spinning (MAS) ssNMR to obtain atomic-level insights into the structure of lipidic fibrils grown under extracellular (pH 7.4) conditions. Small unilamellar vesicles composed of 1-palmitoyl-2-oleoyl-*sn*-glycero-3-phospho-choline (POPC) and -serine (POPS) in an 8:2 ratio (20% POPS) were used. As hIAPP–membrane interactions are sensitive to anionic lipids,^26^ this composition balances physiological relevance with experimental robustness and remains closer to the total anionic lipid content in β-cells (2.5–13.2%)^31^ than typical model systems. Kinetic studies revealed how membrane interaction drives fibril assembly, highlighting the effects of concentration, pH, salt, and temperature on membrane-mediated aggregation. Comprehensive resonance assignments enabled chemical shift analysis, revealing significant differences between lipidic fibrils grown at pH 7.4 and those formed under acidic intracellular conditions (pH 5.3). NMR restraints for the pH 7.4 lipidic fibrils facilitated a high-resolution structure, which was compared to available nonlipidic fibril structures. The core adopts a common fold, suggesting an intrinsic tendency of hIAPP to form this structure, which may extend to in vivo conditions. However, variations in the N- and C-terminal regions highlight the importance of modeling physiological pH and lipid environments in the absence of ex vivo fibril structures.

**Figure 1:**
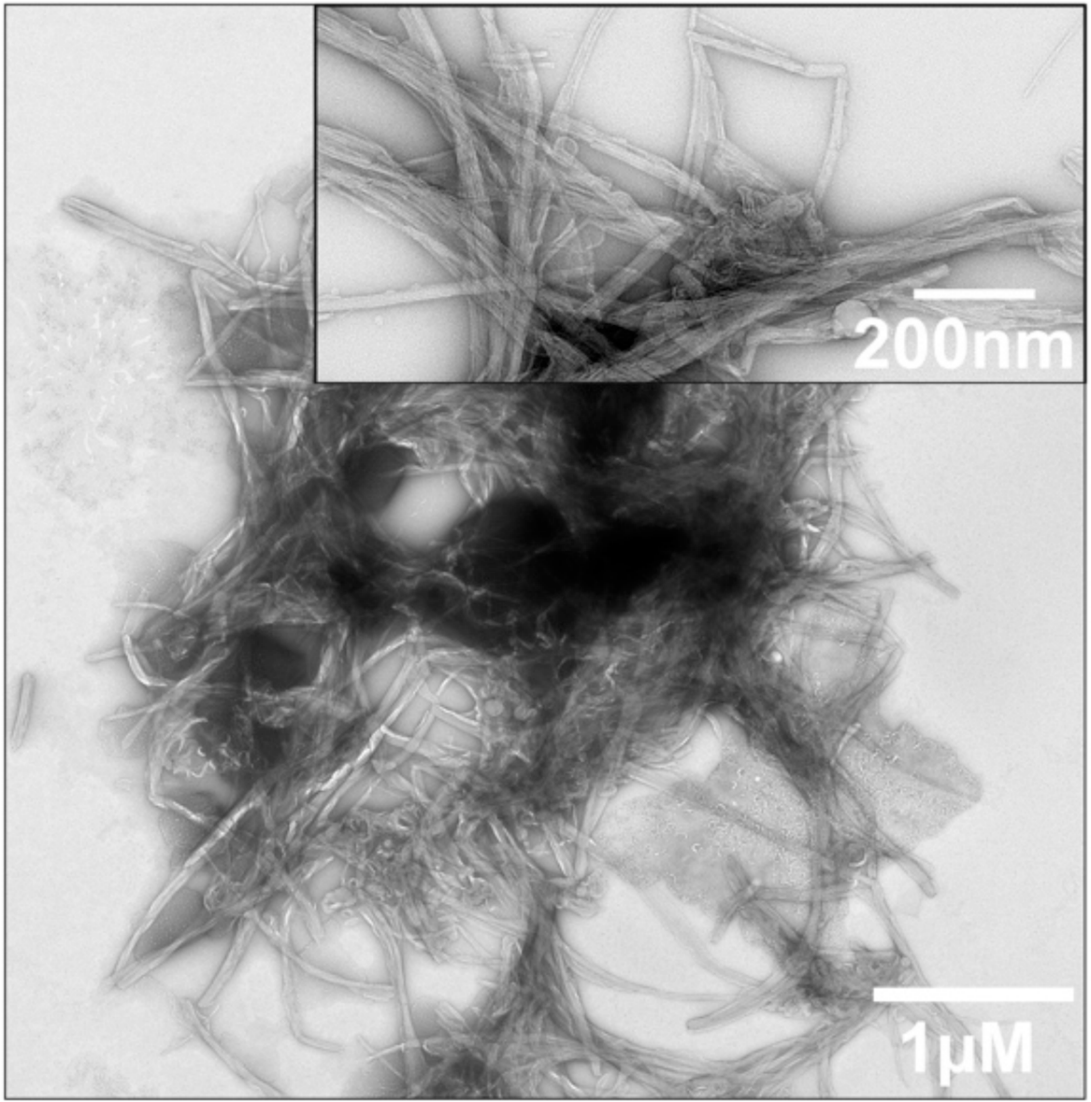
Clumped lipidic fibrils: Negative-stain electron micrograph of fibrils formed by 20 μM hIAPP aggregated in the presence of 100 μM POPC:POPS (8:2) vesicles in 10 mM phosphate buffer (pH 7.4) containing 50 mM NaCl, incubated for 66 h at 37 °C.

## Materials and Methods

### Recombinant Expression and Purification of hIAPP

Recombinant hIAPP was produced as described previously,^32^ with modifications. hIAPP was expressed in BL21­(DE3) as a fusion protein comprising an N-terminal solubilization domain and a C-terminal intein-chitin binding domain (CBD), in minimal medium at 16 °C overnight. For ^15^N/^13^C labeling, ^15^N-ammonium chloride (1 g/L) and ^13^C_6_-glucose (4 g/L) were used as nitrogen and carbon sources. Cells were lysed using a French pressure cell, and the lysate was applied to a chitin column. On-column cleavage of the C-terminal fusion was performed at 4 °C for 3.5 h using 100 mM DTT and 2 M ammonium bicarbonate, releasing the C-terminally amidated hIAPP. The eluate was precipitated at 4 °C with 52% saturated ammonium sulfate, and the resulting pellet was dissolved in 100% HFIP (1,1,1,3,3,3-Hexafluoro-2-propanol) to monomerize the peptide. HFIP was removed under a nitrogen stream and the sample was lyophilized. This HFIP/lyophilization cycle was repeated thrice. The lyophilized peptide was dissolved in V8 digestion buffer (20 mM HEPES pH 8.0, 0.1 mM EDTA (ethylenediaminetetraacetic acid), 2 M urea, 50 mM NaCl, 1 mM DTT) at ≤ 1 mg/mL, and the N-terminal fusion was cleaved with V8 protease (Worthington) on ice for 3 h at a 1:10 (w/w) protease/peptide ratio, followed by shock-freezing in liquid nitrogen. The cleaved peptide was purified by RP-HPLC (Reversed-Phase High-Performance Liquid Chromatography) (JASCO) on a C4 column (Vydac 214TP, 5 μm, 250 × 8 mm) using a 5–95% linear gradient (solvent A: water + 0.1% TFA (Trifluoroacetic acid); solvent B: acetonitrile + 0.1% TFA). Fractions containing hIAPP were confirmed by LC-ESI-MS (Acquity Arc + SQD2, Waters), lyophilized, dissolved in 7 M guanidinium hydrochloride with 1 mM DTT (Dithiothreitol), and repurified by HPLC. Following lyophilization, the peptide was redissolved in 100% HFIP to ensure monomerization, dried under nitrogen, and lyophilized. To form the C2–C7 disulfide bond, the peptide was dissolved in ice-cold water with 5 mM hydrogen peroxide, and oxidation was monitored by LC-MS. In addition, the oxidation state of the peptide was monitored using chemical shift changes observed in solution NMR spectra (Figure S2), as described previously.^32^ The disulfide bridge (C2–C7), conserved across species and essential for IAPP’s biological function, was retained for reasons of physiological relevance.^7^ After complete oxidation, the sample was lyophilized, weighed, and dissolved in an appropriate volume of DMSO-*d*_6_ (Dimethyl sulfoxide) to achieve a final concentration of 1 mM, incubated for 1 h at room temperature, aliquoted, flash-frozen, and stored at – 78 °C.

### Preparation of Vesicles

Small unilamellar vesicles (SUVs) composed of 1-palmitoyl-2-oleoyl-*sn*-glycero-3-phosphocholine (POPC) and 1-palmitoyl-2-oleoyl-*sn*-glycero-3-phospho-l-serine (POPS) were prepared in the desired molar ratio. Stock solutions of POPC and POPS (1 mM) in chloroform were mixed, and the solvent was evaporated under a gentle stream of nitrogen to form a thin lipid film, which was dried under vacuum overnight. The lipid film was hydrated in buffer to a final lipid concentration of 1 mM. Vesicles were prepared in three buffer conditions: 10 mM phosphate buffer containing 50 mM NaCl (pH 7.4), 30 mM acetate buffer (pH 5.3), and 30 mM acetate buffer containing 50 mM NaCl (pH 5.3). The lipid suspension underwent five freeze–thaw cycles, followed by 3–4 cycles of bath sonication (10 min each) until a clear solution was obtained. The vesicle solution was passed through a 0.2 μm syringe filter prior to analysis. To determine the final lipid concentration, an aliquot was diluted into 90% (v/v) methanol-d_4_ (MeOD) containing 0.5% (v/v) dimethylformamide (DMF) as internal reference, and ^1^H NMR spectra were recorded on a Bruker 600 MHz Avance III HD spectrometer (14.1 T). Vesicle size distribution was measured using a DynaPro dynamic light scattering (DLS) instrument (Wyatt Technology) and analyzed with DYNAMICS 6.12.03, yielding an average hydrodynamic diameter of 32 ± 4 nm. Negative-staining transmission electron microscopy (TEM) revealed a size distribution maximum at 22 nm.

### Monomer Consumption Assay

Appropriate volumes of 2 mM ^15^N enriched hIAPP DMSO stock and 1 mM vesicle stock were diluted into precooled (4 °C) buffer containing 5% (v/v) D_2_O to achieve the desired lipid-to-peptide ratio (LPR). The samples were transferred to precooled 3 mm NMR tubes and placed in an NMR spectrometer set to 37 °C, followed by a 5 min incubation to reach temperature equilibrium (dead time, *T*_D_). Time-dependent ^1^H one-dimensional (1D) NMR spectra were recorded at 37 °C (Figure S3), with baseline corrections applied using control samples containing hIAPP alone, vesicles alone, and buffer with 1% (v/v) DMSO-*d*_6_. Monomer consumption was assessed by monitoring the decay in integrated intensities of nonoverlapping hIAPP peaks in the amide, aromatic, and aliphatic regions, while lipid intensity was tracked via the ^1^H signal from the N­(CH3)­3 (choline methyls) in lipid headgroups centered at 3.6 ppm. For samples exhibiting significant monomer loss within 12 h, the decay curves were fitted to a sigmoidal function to extract *T*_50_. At LPR of 5 (acetate buffer without NaCl), a mixed exponential-sigmoidal function was used to account for the initial rapid decay phase. The data shown are representative of at least two independent data sets. All NMR measurements were made on Bruker 600 MHz spectrometers, including the 600 MHz Avance NEO (CPP TCI 600S3 H&F–C/N-D-05 Z ET cryogenic probe), 600 MHz Avance III HD (CP2.1 QCI 600S3 H/F–C/N-D-05 Z XT cryogenic probe), and 800 MHz Avance NEO (CP2.1 TCI 800S6 H/C/N-D-05 Z XT cryogenic probe). ^1^H–^15^N two-dimensional (2D) HSQC spectra were recorded, with 4096 complex points in the ^1^H dimension (spectral width: 20.15 ppm; acquisition time: 126.98 ms) and 64 points in the ^15^N dimension (spectral width: 26 ppm; acquisition time: 15.17 ms). All 1D and 2D NMR data were processed using TopSpin 4.0.8.

### Thioflavin-T Assay

Thioflavin-T (ThT) fluorescence assays were performed in triplicate in a 384 well plate with a final volume of 120 μL per well, containing 20 μM ThT in desired buffer. Low Volume 384-well Black/Clear Flat Bottom Polystyrene NBS Microplates (Corning) were used to minimize surface adsorption effects. To reduce spontaneous aggregation during setup, all solutions and plates were handled in a cold room (4 °C), with hIAPP stock added last. The plates were maintained on ice and promptly transferred to the plate reader following assembly. Each plate was sealed with a transparent adhesive film (Biozym Scientific GmbH) to prevent sample evaporation. Kinetic measurements were performed at 37 °C using a BioTek Synergy Neo2 plate reader, with excitation at 440 nm (bandwidth 20 nm) and emission at 483 nm (bandwidth 20 nm). Fluorescence intensities were baseline corrected and plotted as a function of time. The data presented are representative of at least two independent experiments. Fluorescence curves were fitted to a sigmoidal function to extract the aggregation half-time (*T*_50_), defined as the time at which the signal reaches 50% of its final plateau value.

### Transmission Electron Microscopy

Following 66-h aggregation of 20 μM hIAPP at 37 °C, in the presence and absence of vesicles in 20 mM HEPES buffer (pH 7.4), the samples were kept on ice and prepared for TEM imaging. The samples were applied to a carbon coated copper TEM grid, and stained with 1% uranyl acetate aqueous solution. Imaging was performed at room temperature using a Talos L120C (Thermo Fisher Scientific) electron microscope. For vesicle-only controls, a freshly prepared solution was prepared and imaged following the same protocol.

### ssNMR Spectroscopy

Twenty μM ^13^C,^15^N-enriched hIAPP was aggregated (under quiescent conditions) with 100 μM POPC/POPS (8:2) vesicles in 10 mM phosphate buffer containing 50 mM NaCl (pH 7.4 lipidic fibrils) and 30 mM acetate buffer containing 50 mM NaCl (pH 5.3 lipidic fibrils) for 66 h in flat-bottom glass vials at 37 °C. After incubation, the 6 mL suspension was transferred to 1.5 mL microfuge tubes and ultracentrifuged at 123,662*g* for 1 h at 4 °C using a Beckman Coulter Optima MAX-XP ultracentrifuge with a fixed-angle TLA100.3 rotor. The resulting pellets were resuspended in buffer containing 80 mM Cu-EDTA and subjected to a final centrifugation step. The presence of paramagnetic Cu^2+^ ions enhanced relaxation rates of nearby nuclei, which shortened the recycle delay between measurements from 1.5 to 0.3 s without having any observable impact on the hCANH spectrum of hIAPP fibrils.^33^ The final pellet was then transferred into a 1.3 mm or 1.9 mm MAS rotor for measurements.

All ^1^H-detected spectra, including 2D hNH, hCH, and 3D SPEPS^34^-based hCANH, hCAcoNH, hCOcaNH, hCONH, hCBcaNH, and hCBcacoNH,^35^ as well as GODIST-based hCCH-GODIST and MODIST-based hChhCH-MODIST,^36^ and a 3D lipid-edited HhCH (with a 140 Hz T_2_-filter and 40 ms z-mixing)^37–39^ were recorded on an 800 MHz Bruker Avance III HD spectrometer (18.8 T) equipped with a 1.3 mm MAS HCN probe, operating at 55 kHz MAS. Additionally, ^13^C-detected spectra were recorded, including hCC RFDR^40^ (4.65 ms mixing time) and hCC with a Cβ-Cα hSPEPS^34^ element (1.16 ms mixing time). SPEPS pulse schemes were applied for Cα-N and CO-N transfers, with radio frequency (RF) strength optimized at ∼ 41 kHz for ^13^C and ∼ 14 kHz for ^15^N. The hSPEPS scheme was also used for Cα-CO and CO-Cα transfers, with RF strength set to 1 × MAS frequency, while Cβ-Cα transfer was performed at 68.75 kHz (1.25 × MAS).

For magnetization transfer, hCCH-GODIST utilized a GODIST block^41^ at 0.5 × MAS RF for selective ^13^C–^13^C mixing, while hChhCH-MODIST employed a MODIST block^42^ at 0.25 × MAS RF for efficient ^1^H–^1^H mixing. In hChhCH-MODIST, short cross-polarization (CP) periods (∼250 μs) were used for H–C and C–H transfers to enhance Cα signal intensity, compared to longer CP times. The cooling gas was maintained at 245 K, yielding a sample temperature of 299 K estimated from the water signal externally referenced relative to Sodium trimethylsilylpropanesulfonate (DSS) using ^1^H chemical shift (1.28 ppm) of lipid chain −(CH_2_)– groups. During ^1^H evolution, WALTZ-16 (∼10 kHz) was applied for ^15^N and ^13^C decoupling, and MISSISSIPPI water suppression^43^ was performed for 120–150 ms. Low-power (0.25 × MAS) ^1^H dipolar decoupling during ^13^C and ^15^N evolution was applied using the SW_f_-TPPM scheme.^44^ ^1^H → X and X → ^1^H CP transfers were achieved using linearly ramped (80 → 100%) pulses.

A series of low-MAS 2D hCC DARR^45^ spectra were acquired on a 950 MHz Avance III spectrometer (22.3 T) using a 1.9 mm MAS HCN probe. Data sets were recorded with mixing times of 20, 100, 200, and 400 ms at MAS frequencies of 14.789, 19.444, and 21.111 kHz to minimize peak overlap ambiguities from spinning sidebands. During all ^13^C detection periods, the cooling gas was maintained at 270 K, yielding an estimated sample temperature of 20 °C. High-power ^1^H decoupling was applied during ^13^C acquisition using the SW_f_-TPPM scheme, with a recycle delay of 2.0 s. All spectra were processed using TopSpin 4.0.8 and analyzed with Analysis V3 (CCPNMR, http://www.ccpn.ac.uk/ccpn). The average line widths for ^1^H, ^15^N, and ^13^C nuclei are on the order of 0.5, 2.2, and 1.0 ppm, respectively. A detailed set of parameters is available in the Supporting Information (Table S1). Average chemical shift perturbation (CSP) between pH 7.4 and pH 5.3 fibrils were calculated using Cα, Cβ, and Hα chemical shifts according to the following equation based on Williamson et al.,^46^

$$a\mathrm{verage CSP}=\frac{1}{3}\sqrt{{\left(2.0\times \Delta C\alpha \right)}^{2}+{\left(1.0\times \Delta C\beta \right)}^{2}+{\left(12.9\times \Delta H\alpha \right)}^{2}}$$

### Cyana Structure Calculations

The ssNMR data-driven fibril structure was constructed using CYANA^47^ with 200 structures and 200,000 steps. Backbone torsion angles were incorporated based on TALOS-N predictions, derived from Cα, Cβ, CO, ^H^N, Hα, and H chemical shifts. The quality of TALOS-N predictions was evaluated based on database consensus, with all residues except K1, C2, F23, G24, S28, and N31 meeting confidence criteria. For loop regions, the predicted angles were restricted within ± 3σ of the TALOS-N error estimates, while for β-strand residues, a tighter restraint of ± 1σ was applied. Due to the close similarity of predicted angles for residues 14–27, the dihedral angles of F23 and G24 were adopted from the 6Y1A S-type cryo-EM structure and constrained within ± 30°.^15^ A comparison with structure calculations run using the ambiguous TALOS-N predictions for F23 and G24 is included in the Supporting Information (Section S1 and Figure S19). The fibril was built as a four-layered assembly, with each layer positioned ∼ 4.7 Å apart and containing a single molecule per layer. To enforce conformational uniformity across monomers, an identity restraint function was applied, ensuring consistent dihedral angles between equivalent residues in different layers.^48^ Structural stabilization was further reinforced by defining hydrogen bond constraints along the fibril axis, with O–H bond lengths restricted to 1.8–2.0 Å and O–N bond lengths limited to 2.7–3.0 Å for residues predicted to adopt a β-strand conformation by TALOS-N. A total of 17 unique medium- and long-range distance restraints (Figure S15), identified from 3D hChhCH MODIST and 2D hCC DARR spectra, were incorporated into each subunit for structure calculations. A disulfide bond between C2 and C7 was enforced by restraining the S–S distance between 1.0 and 1.5 Å. Additionally, the distances between Cα atoms of β-strand residues involved in the β-arch motif were constrained within 9–11 Å. Structure calculations yielded a final bundle of the 10 lowest-energy conformers, with a target function of ∼ 5. The resulting ensemble exhibited an average backbone RMSD of 0.87 ± 0.39 Å and heavy atom RMSD of 1.21 ± 0.38 Å over residues A8 to Y37, relative to the mean structure.

## Results and Discussion

### Aggregation Kinetics and Membrane Affinity

Membrane-driven hIAPP aggregation is predominantly governed by secondary nucleation, where new fibrils emerge on pre-existing ones.^17,22^ The process involves three key events: membrane binding, self-assembly, and structural transformation of hIAPP monomers. Figure 2A depicts the depletion of soluble hIAPP monomers, monitored by solution NMR, as a measure of membrane binding and self-assembly, independent of underlying structural transformations. Free monomers produce sharp signals due to rapid tumbling, but binding to slow-tumbling vesicles or incorporation into aggregates leads to signal loss from the interacting residues (Figure S3). Unlike α-synuclein in PD, where the flexible C-terminus remains visible in solution NMR even when bound to vesicles,^49^ all residues in hIAPP are affected by reduced molecular tumbling, leading to uniform signal loss upon membrane association. This decrease in signal intensity, referred to as *monomer consumption*, was tracked over time. In the absence of lipids, monomer consumption due to self-assembly occurs with a *T*_50_ (time for half of the soluble monomer population to deplete) of 2.65 ± 0.03 h. The addition of vesicles at an LPR of 5 markedly accelerates this process, reducing *T*_50_ to 0.53 ± 0.01 h. This significant acceleration reflects both rapid initial membrane binding and subsequent self-assembly of hIAPP monomers. Similarly, at pH 5.3, the presence of vesicles enhances consumption kinetics, with a nearly 14-fold reduction in *T*_50_. Low pH is known to slow fibril formation both in solution and in membrane-associated contexts.^28^ In secretory granules, the combined effects of low pH, zinc, and insulin work together to suppress hIAPP fibrillization—factors that were not included in our in vitro model.^29^

**Figure 2:**
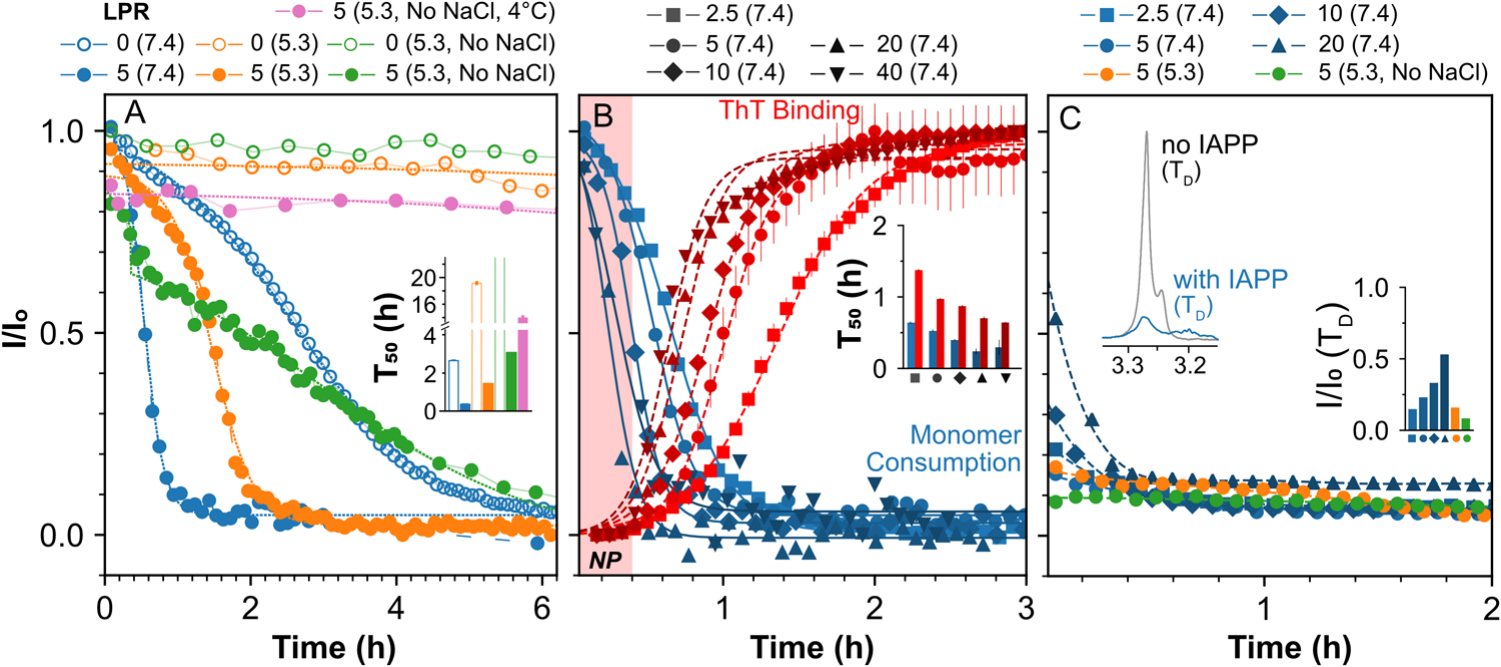
Membrane-mediated aggregation kinetics: (A) Time-dependent decay of normalized NMR peak intensity of soluble hIAPP monomers at different lipid-to-peptide ratios (LPRs) and buffer conditions (see legend). All curves except LPR 0 (pH 5.3, no NaCl) were fitted to a sigmoidal function to extract *T*_50_ values, shown as a bar plot with fitting errors (inset). The LPR 0 (pH 5.3) data set was excluded due to negligible decay. For LPR 5 (pH 5.3, no NaCl), a mixed exponential–sigmoidal function was used to capture the initial rapid decay. (B) NMR-derived monomer consumption curves (blue) and normalized ThT fluorescence intensities (red) for various LPRs (see legend). Both data sets were fitted to sigmoidal functions to extract *T*_50_ values (bar plot with fitting errors in inset). The nucleation phase (NP, ThT *I*/*I*_0_ < 5) is shaded in red. A 12-h time scale comparison of panels A and B is provided in Figure S4. (C) Normalized NMR peak intensity of the lipid choline signal (3.25 ppm) over time, fitted to a polynomial function, for each LPR and buffer condition. Inset: lipid peak decay during the measurement dead time (*T*_D_, 5 min), and change in choline signal upon addition of 20 μM hIAPP.

At some point during aggregation, specific segments of hIAPP monomers undergo structural transformation into β-strands, assembling into highly ordered cross-β fibrillar structures. ThT fluorescence, an indicator of β-strand formation, exhibits a nonlinear decrease in *T*_50_ values as LPR increases from 2.5 to 40, eventually approaching saturation (Figure 2B). This aligns with findings by Elenbaas et al.,^17^ who showed that nucleation, reflected in *T*_50_, is independent of hIAPP density on the membrane at high LPR, with a single membrane-bound monomer being sufficient to initiate fibril formation. More importantly, ThT *T*_50_ values remain consistently higher than those for monomer consumption, highlighting a lag between loss of soluble monomers and formation of β-strands. During the first 20 min, termed the *nucleation phase*, ThT fluorescence remains largely unchanged (*I*/*I*_0_ < 5), while a significant fraction of soluble monomers is already consumed—approximately 20%, 25%, 45%, and 90% at LPRs of 2.5, 5, 10, and 20, respectively. A similar trend is observed under pH 5.3 conditions (Figure S4). hIAPP monomers adopt α-helical conformations upon membrane binding,^50–52^ and unless they transition into β-strand-rich structures, they remain undetectable by ThT fluorescence assay. While some studies suggest that membrane-bound monomers directly convert into fibrils,^17^ others supports the formation of oligomeric intermediates.^22^ Given that these assays cannot distinguish between helical monomers and β-strand-deficient oligomers, the nucleation phase may include both populations.

The transient nature of the membrane-bound state complicates experimental characterization of the binding mechanism. Evidence suggests that the N-terminal half of hIAPP mediates membrane attachment, with certain segments adopting a helical conformation, while the C-terminal half drives fibril formation.^50,52–54^ At low pH, with H18 carrying an additional positive charge, low ionic strength conditions enhance membrane association.^26,28^ This is evidenced by an immediate ∼ 15% loss in hIAPP monomer population in acetate buffer lacking 50 mM NaCl at LPR of 5 (Figure 2A). To suppress subsequent self-assembly, we performed the same measurements at 4 °C. A similar initial ∼ 15% monomer loss was observed, followed by a prolonged plateau phase with no further depletion (Figure 2A), effectively decoupling membrane binding and self-assembly events. NMR signals from backbone amide groups (NH) decay uniformly across all residues during this plateau phase, indicating full peptide interaction with the membrane (Figure S5).

The impact of hIAPP membrane association is particularly evident in the decay of the NMR signal originating from the choline moiety of the POPC headgroup (Figure 2C). When exposed to the water–lipid interface, this moiety retains sufficient flexibility to produce a distinct peak in ^1^H 1D spectrum. Upon addition of hIAPP, the signal is almost entirely lost within the dead time of the measurement (*T*_D_ = 5 min). A similar reduction was observed for the hydrogens in the lipid acyl chains (Figure S3), indicating that this effect is not caused solely due to structural ordering of the lipid headgroup driven by electrostatic interactions. hIAPP binding to negatively charged vesicles (due to the presence of 20% POPS) likely weakens the long-range electrostatic repulsion between vesicles. This could promote vesicle clustering or aggregation, thereby reducing the rotational correlation time of the vesicles and resulting in a rapid loss of NMR signal. This effect is consistent across both low and high pH conditions and is also observed in the absence of salt (Figure 2C).

### Assignment and Secondary Structure

Lipidic hIAPP fibrils, aggregated for 66 h in the presence of POPC:POPS(8:2) vesicles under quiescent conditions in phosphate buffer at pH 7.4, were analyzed using ssNMR spectroscopy. An LPR of 5 was selected for ssNMR sample preparation, as this ratio allows sufficient hIAPP packing into MAS rotors, enhancing sensitivity—particularly important for low-signal experiments such as 3D T_2_-filtered measurements. Kinetic studies indicate that, at this ratio, aggregation is primarily driven by lipid membranes (Figure 2B). Whether further acceleration of aggregation observed at higher LPRs results in structural differences remains to be determined but is even more difficult than the study presented here, because the signal-to-noise of the protein resonances will be lower. The presence of lipids in the fibril pellet was confirmed by both ^1^H and ^31^P NMR spectroscopy, which showed characteristic lipid signals (Figure S6). Quantitative analysis further indicated that virtually all lipids were present in the pellet, with no detectable lipid remaining in the supernatant. Representative N–C and H–C correlation spectra are shown in Figure S7. Sequential assignment of ^13^C- and ^15^N-labeled hIAPP fibril samples was achieved using dipolar-based, ^1^H-detected C–N–H 3D correlation spectra (see Materials and Methods for details). Sequential assignment linked backbone N–H pairs of residues with neighboring Cα, Cβ, or Carbonyl carbons, enabling step-by-step assignment of backbone resonances (Figure S8). To complement this data and resolve ambiguities caused by peak overlap, ^1^H-detected C–C–H 3D and ^13^C-detected C–C correlation 2D spectra were also acquired. These aided side-chain carbon assignments and helped assign hydrogen atoms in both the backbone and side chains. The Hα chemical shifts obtained were crucial for TALOS-N-based secondary structure prediction.^55^

Most amino acids yielded a single set of chemical shifts, except V17 which exhibited two similar sets (Figure 3A and Table S1). Likewise, I26 yielded two sets of chemical shifts for its Cβ, Cγ_2_, and Cδ_1_ carbons. These possible side-chain rotamers of I26 are clearly distinguishable in the DARR-based C–C 2D spectrum (Figure S9). DARR spectra aid in identifying long-range through-space contacts, playing a key role in confirming the assignments of H18, F15, F23, and Y37, whose aromatic side chains were identified through intraresidue connectivity (Figure S10). Additionally, R11 carbons were assigned based on Cζ cross-peaks (Figure S10C), while Cδ cross-peaks in Q10 provided a clear assignment. C7 was identified based on its high Cβ chemical shift, indicative of its involvement in a disulfide bond.^56^ A sequential link was observed for residues T4, A5, and T6 (Figure S8). Notably, A5 exhibited unusually high Cβ chemical shifts, likely resulting from the formation of a disulfide loop between C2 and C7. Carbon chemical shifts of C2 were assigned based on previously reported chemical shift values for oxidized C2.^7^ K1 and N3 remained unassigned due to a lack of unambiguous cross-peaks.

**Figure 3:**
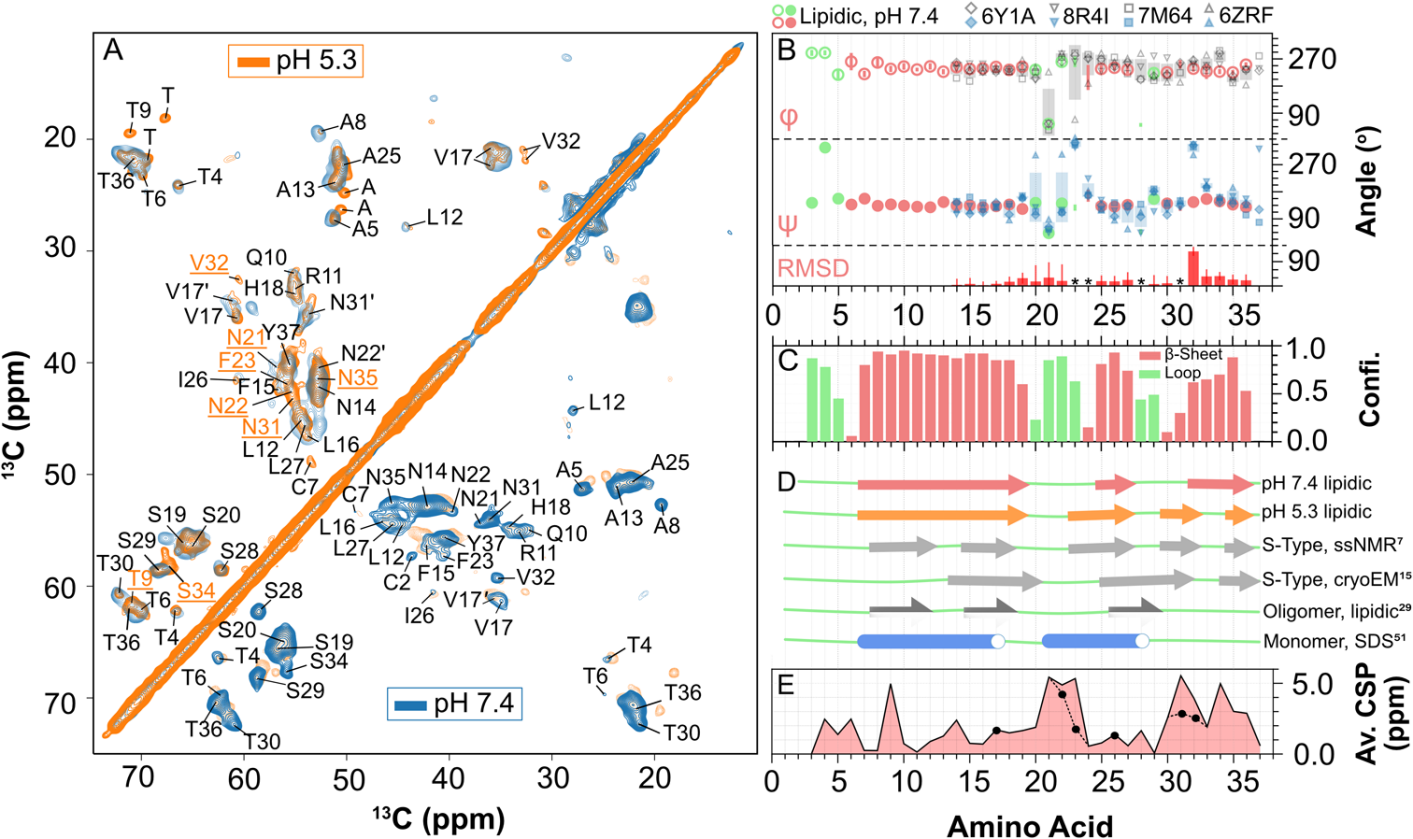
Secondary structure comparison: (A) 2D hCC SPEPS spectra of lipidic hIAPP fibrils grown at pH 7.4 (blue) and pH 5.3 (orange), showing C–C cross-peaks in the aliphatic region. Residues exhibiting significant peak shifts at pH 5.3 are underscored. Alternative peak sets are marked with a prime symbol. (B) TALOS-N predicted φ (open circles, top panel) and ψ (filled circles, middle panel) dihedral angles (360° scale) and associated errors for pH 7.4 lipidic fibrils, compared to residues 14–36 in various S-type cryo-EM structures (see legend). Cryo-EM angle distributions are shown as shaded bars representing the error range around the average angle from four structures per residue. The bottom panel shows root-mean-square deviation (RMSD) of torsional angles between pH 7.4 fibrils and the averaged cryo-EM S-type structures. Residues with low-quality predictions are excluded and marked with asterisks. An enlarged version of panel B is available as Figure S11. (C) TALOS-N prediction confidence for secondary structure elements in pH 7.4 lipidic fibrils. (D) Comparison of β-strand locations (solid arrows) across lipidic fibrils (this study), nonlipidic S-type fibrils (ssNMR and cryo-EM), lipid-bound oligomers in nanodiscs, and monomeric states in SDS micelles. Antiparallel β-strands in oligomers are shown with half a rrows; helices in monomers are represented by blue cylinders. (E) Average chemical shift perturbations (CSPs) between pH 7.4 and pH 5.3 lipidic fibrils. Filled black circles indicate average CSPs for alternative chemical shift sets observed in specific residues at pH 5.3.

TALOS-N, an artificial neural network-based program, predicts backbone torsion angles and secondary structure propensities using H, N, and C chemical shifts. We utilized a complete chemical shift set (Cα, Cβ, Co, Hα, ^N^H, and N_H_; see Table S2) for both pH 7.4 and pH 5.3 lipidic fibrils. Figure 3B shows the backbone dihedral φ and ψ angles predicted by TALOS-N, along with the secondary structure propensity for each residue in pH 7.4 lipidic fibrils (Figure 3C). Three regions, with confidence values greater than 0.5, were identified as β-strands involving residues C7–S19 (β-1), A25–L27 (β-2), and V32–Y37 (β-3). These β-strands are connected by loops: S20–G24, linking β-1 to β-2, and S28–N31, linking β-2 to β-3. The predicted secondary structural elements are summarized as a cartoon in Figure 3D. Unlike most cryo-EM-based structures, which report residues 1–12 as disordered, the N-terminal region in pH 7.4 lipidic fibrils adopts an extended β-strand conformation starting from C7. Notably, all previous ssNMR-based structures of nonlipidic fibrils also feature extended β-strands toward the N-terminus.^6–8^ Among these, a recent ssNMR study by Suladze et al.^7^ reported a S-Type structure of nonlipidic hIAPP fibrils (PDB ID: 6Y1A)^15^ (Figure 3D). This S-Type fold is among the most commonly observed structural motifs in hIAPP fibrils and has been reported in five independent studies, including cryo-EM structures by Gallardo et al. (PDB ID: 6ZRF),^11^ Cao et al. (Polymorph TW3, PDB ID: 7M64),^13^ and Valli et al. (PDB ID: 8R4I).^9^ The chemical shifts reported by Suladze et al. are compared with those of the lipid-associated fibrils in Figure S12.

Figure 3B also presents the φ and ψ angles for residues 14–37 in the S-Type cryo-EM structures. The angle distribution is generally narrow, except for residues 20–24 in the loop connecting β-1 to β-2, where deviations arise due to unique dihedral angles in 6ZRF. The remaining three structures align well with TALOS-N predictions for pH 7.4 lipidic fibrils in this loop region. V32 and S34 exhibit a significant deviation from the average of the four cryo-EM structures (RMSD > 2σ, Figure 3B). Unlike residues 20–24, which show variability across cryo-EM structures, V32 and S34 have nearly identical angles, making their high RMSD values particularly significant. In cryo-EM structures, the unusual ψ angle of V32 places it in the right-handed α-helix region of the Ramachandran plot—an essential feature for adopting an S-Type fold. In contrast, V32 dihedral angles in pH 7.4 lipidic fibrils fall within the β-strand region, similar to its neighboring residues, highlighting a structural deviation from the S-Type fold observed in nonlipidic fibrils.

### Comparison with pH 5.3 Lipidic Fibrils

A low pH environment markedly influences the membrane-mediated aggregation kinetics of hIAPP, leading to lipidic fibrils with pronounced peak shifts, as seen in the C–C spectrum in Figure 3A. While many assignments align with those of pH 7.4 lipidic fibrils, several residues display unique chemical shifts, and additional assignments were obtained from ^1^H-detected 3D experiments. Residues N22, F23, N31, and V32 exhibit two distinct chemical shift sets at pH 5.3, similar to the multiple conformers previously noted for V17 and I26 at pH 7.4 (Table S3). Additionally, unique chemical shifts were detected for two threonine and two alanine residues, though their precise sequence positions remain undetermined. These findings suggest that pH 5.3 lipidic fibrils exhibit structural heterogeneity, with multiple residues adopting alternative conformations. Protein fibrils are typically composed of more than one molecule per layer, commonly referred to as protofilaments.^57^ Structural heterogeneity in amyloid fibrils can arise from variations in structural fold of protofilaments and/or differences in how protofilaments interact within the fibril layer or stack along the fibril axis. Further details on the protofilament architecture of hIAPP fibrils are provided in a subsequent section.

In addition to heterogeneity, substantial chemical shift perturbations (CSPs) at low pH were observed in T9, N21, N22, S34, N35, and the dominant conformers of F23, N31, and V32. Figure 3E shows average CSPs between pH 5.3 and pH 7.4 fibrils, calculated using Cα, Cβ, and Hα shifts—nuclei least affected by pH-induced hydrogen bonding changes. These changes primarily affect the loop region (S20–G24) linking β-1 to β-2, as well as the C-terminal region. Accordingly, TALOS-N predicts two short β-strands in the C-terminal region (residues 30–32 and 35–36) in pH 5.3 lipidic fibrils (Figure S13), in contrast to the single β-strand observed in pH 7.4 lipidic fibrils (Figure 3D). In addition, β-2 extends two residues toward the N-terminus in pH 5.3 lipidic fibrils with both *F*23 and G24 being part of it. On the other hand, the N-terminal region remains similar in both cases.

Although secondary structure similarity does not guarantee the same tertiary fold, the C-terminal secondary structure of pH 5.3 lipidic fibrils closely resembles that of pH 5.3 nonlipidic fibrils reported by Suladze et al., suggesting a shared S-type fold (Figure 3D). This raises the question of whether the observed chemical shift differences are simply a pH driven effect. However, buffer exchange of preformed pH 5.3 lipidic fibrils with pH 7.4 phosphate buffer did not alter their H–C–N and C–C spectra (Figure S14), confirming that these differences do not result from reversible pH-dependent effects. Instead, they reflect a distinct fold stabilized during slower aggregation kinetics at low pH.

Importantly, aggregation kinetics alone do not always dictate structural differences. For example, fibrils formed in pH 5.3 acetate buffer without 50 mM NaCl exhibited biphasic monomer consumption kinetics (Figure 2A), distinct from those formed with salt. Yet, their spectra were nearly identical to fibrils grown with NaCl (Figure S14), indicating that despite kinetic differences, the final fibril structures remained the same. This further supports that the alternative fold in pH 5.3 lipidic fibrils arises from a specific aggregation pathway favored by phospholipids and low-pH conditions, rather than being a kinetic artifact or a direct pH effect.

### Structure of Lipidic Fibrils

The structure of pH 7.4 lipidic hIAPP fibrils was determined using CYANA,^58^ incorporating ssNMR-derived structural restraints and prior knowledge of amyloid fibril architecture. It uses backbone dihedral angles and secondary structure elements predicted by TALOS-N. Crucially, it uses 17 unique long-range (|*i*–*j*| > 2) and 24 short-range (|*i*–*j*| = 2) inter-residue contacts identified in 2D and 3D spectra via H–H and C–C through-space magnetization transfers (Figure S15). Lastly, the structure includes the characteristic cross-β arrangement, with parallel in-register (PIR) β-sheets spaced ∼ 4.7 Å apart. The final structure, as shown in Figure 4A, accurately represents both local conformational preferences and the supramolecular organization of fibrils that satisfies all experimentally derived dihedral and distance restraints. Eight out of ten lowest-energy conformers exhibit a subtle twist in their β-sheets, running along the fibril axis (Figure 4B). Although no experimental restraints confirm this twist in pH 7.4 lipidic fibrils, most amyloid fibrils are known to adopt a left-handed twist.^57^ At the same time, the possibility of flat β-sheets, observed in the two lowest-energy conformers, cannot be ruled out (Figure 4C). Note that the ensemble of structures shown in Figure 4B,C represents the remaining flexibility allowed by the experimental restraints, which, due to the lack of long-range restraints in the flanking regions, results in excursions of the N- and C-terminal regions. Although the structure is represented as a single protofilament, the presence of additional protofilaments remains likely; however, no interfilament interface could be unambiguously identified.

**Figure 4:**
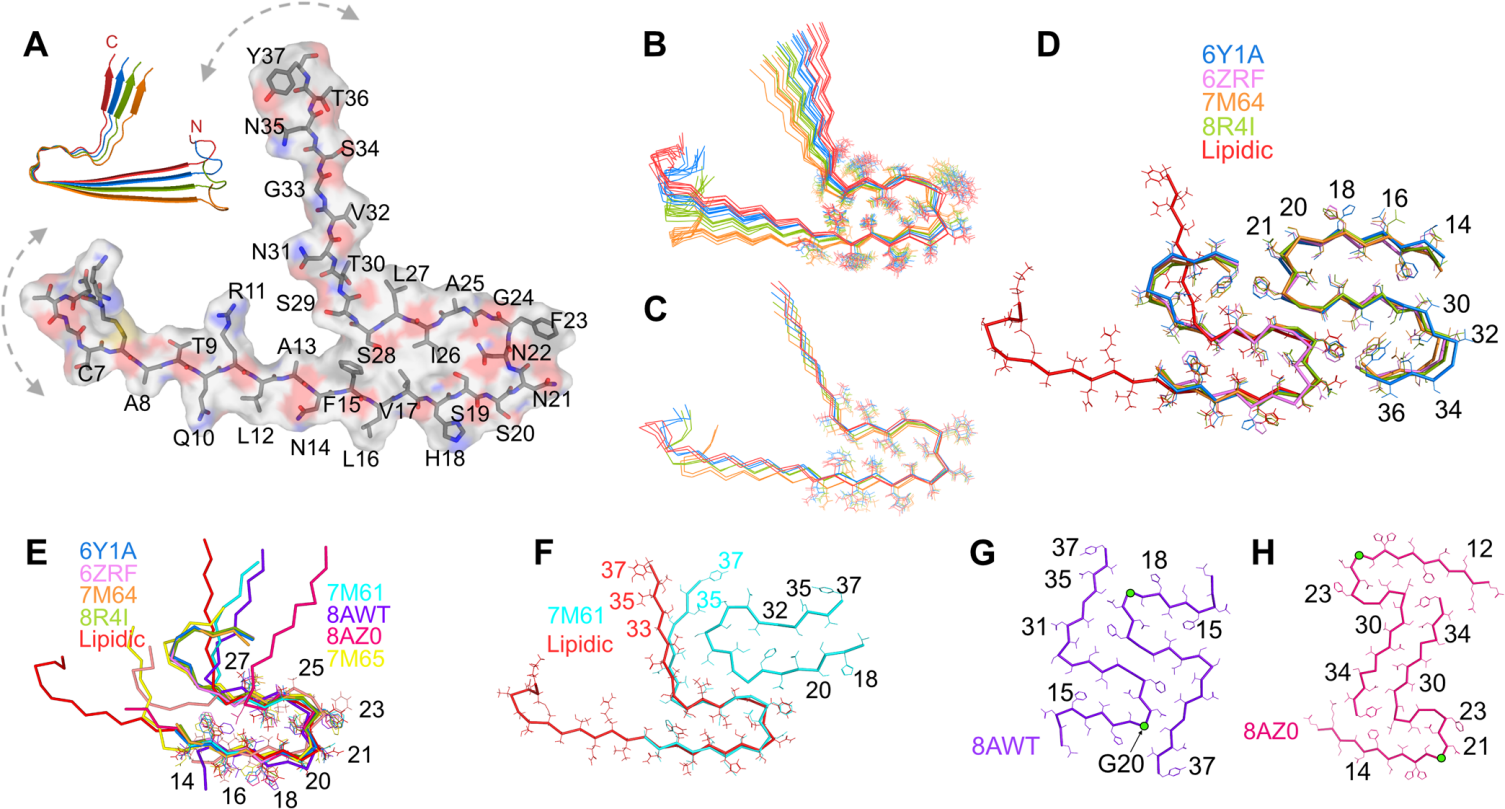
Structure of pH 7.4 lipidic fibrils: (A) Surface-filled structure of a single protofilament representing one molecular layer from the lowest-energy conformer of pH 7.4 lipidic fibrils, built using CYANA. Color-coded by atomic partial charge (blue: positive, red: negative). Inset: stack of four monomers displaying a cross-β structure. Dotted double arrows indicate potential flexibility arising from limited long-range restraints in the flanking N-terminal (residues 1–12) and C-terminal (residues 31–37) regions. (B, C) Ten lowest-energy conformers generated from CYANA calculations, each consisting of four protofilaments arranged in a cross-β architecture. Individual monomers are shown in different colors for clarity. Most conformers exhibit a subtle β-sheet twist (B), while two display a flatter β-sheet arrangement (C). (D) Alignment of a lipidic fibril layer with a nonlipidic S-type fibril composed of two protofilaments in a C2-symmetric arrangement. (E) Superposition of the lipidic protofilament with eight nonlipidic fibrils, aligned on the conserved CF1 fold (residues 14–27); side chains shown as sticks. (F) TW1 polymorph (PDB 7M61) featuring a CF1-type protofilament (aligned with the lipidic protofilament) interfacing with a CF2 protofilament. Molecular layers from (G) early stage and (H) mature S20G hIAPP fibrils. PDB accession codes are color-coded to match the corresponding protofilaments.

Figure 4D presents a comparative alignment of a single molecular layer from lipidic fibrils with all reported S-type structures of hIAPP. Backbone RMSD analysis reveals a conserved fold for residues 14–27, with lipidic fibrils exhibiting the lowest RMSD (0.868 Å) to 6Y1A, followed by 8R4I (0.935 Å) and 7M64 (1.007 Å), and 6ZRF (1.232 Å). The increased RMSD in 6ZRF arises primarily from distinct dihedral angles adopted by loop region residues (N21–G24), despite preserving side-chain orientation (Figure S16). This likely reflects cryo-EM structures derived from maps resolved between 3.0 and 4.2 Å, producing slightly different loop conformations while preserving the β-arch connecting β-strand 1 and 2 in S-type structures. A β-arch, found in amyloid fibrils, differs from β-hairpins in globular proteins. It retains a hairpin-like shape but lacks backbone hydrogen bonding between consecutive strands, relying instead on side-chain interactions and intermolecular hydrogen bonds for stability. Our ssNMR data revealed well-defined connections between residues 13–29, 15–26, 17–26, 17–28, 19–26, and 22–26 supporting formation of this β-arch in lipidic fibrils.

#### Conserved Core

A key feature of our structure is the similarity of residues ^14^NFLVHSSNNFGAIL^25^ in lipidic fibrils to the highly conserved core fold, designated as Core Fold 1 (CF1) by Cao et al. (Figure 4E).^13^ To date, three protofilament folds have been identified in wild-type nonlipidic hIAPP fibrils. The CF1 fold is the most prevalent structural motif present across a range of polymorphs, including non-S-type structures such as 7M61 and 7M65. It is also found in both early stage (8AWT) and mature fibrils (8AZ0, 8AZ4, 8AZ6, 8AZ7, and 6ZRR) of the S20G mutant. The primary structural difference among CF1-containing fibrils lies in the orientation of residues 28–37. In S-type structures, V32 and G33 play a crucial role in facilitating the folding of the chain upon itself, leading to a compact S-shaped topology where the side chains of residues in β-3 (N35 and Y37) interact closely with N31 and L27 in β-2 (Figure 4D). However, in lipidic fibrils, both V32 and G33 are part of an extended β-strand. As expected, our ssNMR measurements did not detect the long-range contacts between residues in β-2 and β-3 characteristic of the S-type fold. Figure 4F presents a molecular layer of the most abundant polymorph in patient seeded wild-type hIAPP fibrils, 7M61, in which CF1 containing chain A is aligned with the lipidic protofilament. In lipidic fibrils, this “L”-shaped structural fold is supported by strong cross-peaks observed between the backbone and side chain atoms of L27 and T30 via C–C and H–H transfers (Figure S17). A similar L- fold has been observed in two S20G fibril polymorphs 8AWT (Figure 4G) and 8AZ0 (Figure 4H) as well.

#### Extended N-Terminal Region

One of the major distinctions between our ssNMR-based protofilament structure of lipidic fibrils and cryo-EM structures of nonlipidic fibrils is the length of the N-terminal β-strand. Most cryo-EM structures report residues 1–12 as disordered, attributed to poorly resolved maps in this region. Although extended N-terminal β-sheets have been reported in some cryo-EM structures, they appear to be less common. For instance, the lowly populated chain A in TW4 polymorph of seeded wild-type hIAPP fibrils (7M65) adopts a CF1 fold with residues 7–12 forming a β-strand, connected to β-2 (14–19) through a kink at A13 (Figure 4E). Similarly, the L-fold polymorph (8AZ0) in S20G mutant fibrils exhibits an extended N-terminal β-strand spanning residues 9–20 without a kink (Figure 4H).

#### Potential Structural Reorganization in the Early Stages

The presence of an extended β-strand in the N-terminus may reflect a structural reorganization that occurs during the early stages of membrane-mediated aggregation. The NMR-based structure of monomeric hIAPP in SDS micelles reveals two α-helices spanning C7–V17 and N21–S28 (Figure 3D).^52^ In contrast, oligomeric hIAPP in lipid nanodiscs adopts two β-hairpin motifs, linking three antiparallel β-strands (A8–L12, F15–H18, and I26–S29, Figure 3D).^30^ Similar antiparallel β-strands have been identified in Amyloid-β^59^ and α-Synuclein oligomers.^60–62^ If the lipid nanodisc-bound hIAPP oligomer is on the pathway to lipidic fibrils, a structural shift is required—a 90° rotation of antiparallel β-strands into parallel β-strands, connected via β-arches, a hallmark of amyloid fibrils. The N-terminal half of hIAPP alone must transition from a helical motif in monomers, to two antiparallel β-strands in oligomers linked via a β-hairpin, to a single parallel β-strand in lipidic fibrils (Figure 5). Such transformations would require breaking and reforming hydrogen bonds, introducing high energy barriers that may stabilize potentially toxic oligomeric states. Recently, Sant et al. revealed the structure of an antiparallel β-hairpin motif in a toxic α-synuclein tetramer on the pathway to lipidic fibrils.^62^ These findings show that the hairpin-to-arch transition is crucial for fibril elongation and may represent a common mechanism for membrane-mediated amyloid assembly.

**Figure 5:**
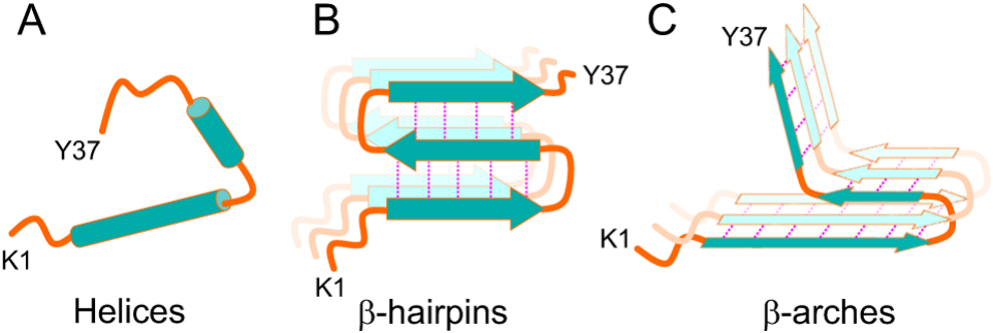
Potential structural reorganization. (A) hIAPP monomers containing helical segments (green cylinders) in SDS micelles (Nanga et al.^52^). (B) Oligomeric β-hairpin motifs with intramolecular hydrogen bonds formed by antiparallel β-strands (green arrows), observed by Rodrigues et al.^30^ in lipid nanodiscs. (C) Lipidic fibrils at pH 7.4 containing β-arch motifs formed by parallel-in-register β-strands. Intra- and intermolecular hydrogen bonds in β-hairpin and β-arch structures are shown as pink dotted lines. If (A) and (B) represent intermediates on pathway to lipidic fibrils in (C), structural reorganization such as a hairpin to arch transition may play a critical role in fibril elongation and the stabilization of intermediate states.

#### Protofilament Architecture

Most amyloid fibrils contain multiple protofilaments per layer,^57^ with two-protofilament arrangements being the most common in nonlipidic hIAPP fibrils (Table S4). Our structure features a single stack of protofilaments, akin to those observed in L1A lipidic fibrils of α-Synuclein^63^ and L2/L3 lipidic fibrils of Aβ40.^64^ However, this structure does not exclude the possibility of a filament–filament interface. Segments 7–13 and 29–37 appear to require additional stabilization, which in the absence of steric zippers, likely arises from protofilament interfaces, peptide contacts, or lipid interactions. Such interfaces may involve side chains from only a few residues, resulting in sparse contacts—as seen in L2–L3 lipidic fibrils of Aβ40—or even no detectable protein–protein contacts, as observed in L2A lipidic fibrils of α-Synuclein and L2–L2/L3–L3 lipidic fibrils of Aβ40.^63,64^ Nonlipidic hIAPP fibrils, including that of S20G mutant, contain either homotypic (e.g., S-type fold, Figure 4D) or heterotypic protofilaments (e.g., Figure 4F). In pH 7.4 lipidic fibrils, a single set of chemical shifts suggests only one protofilament fold, though a homotypic C2 symmetric arrangement remains possible. Ambiguous long-range contacts between S34 and residues N22:N14:A8:C7:N35 as well as S20:S19 with residues N35:A27:G33 partially align with the L-type C2 symmetric interface observed in 8AWT (Figure 4G). Side chains of N31 and Y37 were found to be well-structured, suggesting their involvement in a protein–protein interface, potentially similar to that observed in 8AZ0 (Figure 4H). However, further experimental evidence including additional unambiguous interprotofilament contacts is needed to confirm such an arrangement. Several resolved signals in the C–H plane of a 3D T_2_-filtered z-mixing HhCH spectrum on pH 7.4 lipidic fibrils revealed specific lipid contacts with aromatic and aliphatic residues in the N-terminal region—including F15, L16, L12, and A8—supporting lipid-induced stabilization of the extended N-terminal β-sheet (Figure S18).

The hydrophobic environment provided by phospholipids, absent in previously reported hIAPP structures, may favor a single protofilament per molecular layer, as seen in lipidic fibrils of α-Synuclein and Aβ40. At the individual fibril layer or residue scale, thermodynamic stability, calculated using Eisenberg/Sawaya method,^57^ appears similar across structures with the same number of protofilaments per layer (Table S4). However, stability increases with additional protofilaments, leading to higher mass per unit length, a hallmark of fibril maturation.^10^ This progressive protofilament accumulation may be suppressed in lipidic environments, potentially explaining the lower abundance of fibrils with more than two protofilaments in patient-derived samples of Amyloid-β, Tau, and α-Synuclein. In both lipidic α-synuclein and lipidic Aβ fibrils, lipids interact with specific hydrophobic and charged surfaces, stabilizing fibril morphology in the absence of classical protein–protein interfaces. Similar lipid interactions in hIAPP fibrils may shield exposed fibril surfaces, supporting single-protofilament architectures and reflecting a shared principle of lipid-mediated amyloid assembly.

## Conclusions

Our ssNMR-based structure of hIAPP fibrils, formed in the presence of lipid membranes and under physiological pH, provides an in vitro model of amyloid aggregates relevant to T2DM. Such models are essential for bridging the gap between structural studies and disease pathology and form a basis for the study of early oligomeric aggregates. Beyond the established influence of pH on aggregation kinetics and morphology, we show that fibrils formed at intracellular (pH 5.3) and extracellular (pH 7.4) conditions exhibit distinct structural signatures, particularly in the C-terminal region. Additionally, pH 5.3 lipidic fibrils display structural heterogeneity, whereas their extracellular counterparts adopt a distinct L-shaped protofilament fold. Whether these pH-dependent structural differences contribute to toxicity and link the aggregation site to pathogenicity remains to be explored. Our structure includes a single protofilament per fibril layer in pH 7.4 lipidic fibrils, and a homotypic C2-symmetric arrangement remains a viable possibility. The core segment (^14^NFLVHSSNNFGAIL^25^) adopts the CF1 fold, a β-arch motif also found in wild-type and S20G mutant hIAPP nonlipidic fibrils. The lipidic structure confirms this thermodynamically favored fold under physiological conditions, suggesting its relevance in disease. Our structure reveals an extended N-terminal β-strand, highlighting the advantage of ssNMR in resolving this region, which often remains undetected in cryo-EM. Membrane-mediated aggregation requires significant structural reorganization, transitioning hIAPP from a helical monomer to β-hairpin-rich oligomers with antiparallel β-strands, and ultimately to β-arches in fibrils forming parallel-in-register β-sheets. The hairpin-to-β-arch transition, previously identified as crucial for fibril elongation, may introduce energy barriers that stabilize toxic oligomeric intermediates, which are increasingly recognized as key contributors to hIAPP cytotoxicity.

## Supplementary Material



## Notes

TEM images of vesicles and nonlipidic fibrils, ^15^N–^1^H HSQC spectra of hIAPP with and without disulfide bond; ^1^H NMR spectra depicting monomer consumption; monomer consumption and ThT curves over 12 h in pH 7.4 and 5.3; ^15^N–^1^H HSQC spectra depicting vesicle binding; ^1^H and ^31^P NMR of lipidic fibrils; overlay of 2D ^15^N–^13^C skyline projection of 3D hCANH and 3D hCBcaNH spectra of pH 7.4 lipidic fibrils with peak labels; 2D hCH spectrum of pH 7.4 lipidic fibrils with peak labels; strip plots of 3D hCANH (blue), hCAcoNH (orange), and hCBcaNH (green) spectra of pH 7.4 lipidic fibrils showing sequential assignment; selected regions of the 2D hCC-DARR spectra highlighting side-chain assignments of R11, F15, H18, F23, I26, and Y37 in pH 7.4 lipidic fibrils; TALOS-N prediction confidence for secondary structure elements and dihedral angles in pH 7.4 and 5.3 lipidic fibrils; chemical shift comparison with the nonlipidic fibrils; overlay of 2D hCC SPEPS spectra and 2D C–N projections from 3D hCaNH spectra depicting impact of buffer exchange; long- and short-range inter-residue contacts mapped onto the protofilament structure of pH 7.4 lipidic fibrils; ten lowest-energy conformers generated from CYANA calculations for pH 7.4 lipidic fibrils; dihedral angles of loop region residues extracted from four distinct S-type nonlipidic hIAPP fibril structures; overlay of 2D H–C planes extracted from 3D hCCH GODIST and 3D hChhCH MODIST spectra highlighting key long-range contacts; 2D hCC DARR spectrum of pH 7.4 lipidic fibrils recorded with a 300 ms mixing time highlighting key long-range contacts; 2D projections of 3D lipid-edited HhCH spectrum showing water–protein and lipid–protein contacts; CYANA structure calculations with ambiguous TALOS-N predictions (PDF)
