# Supplementary material for: The Structure of Human IAPP Fibrils Reflects Membrane and pH Conditions
Source: J Am Chem Soc. 2025 Aug 1;147(32):28943–54. doi: 10.1021/jacs.5c06971 (PMC12356532; doi:10.1021/jacs.5c06971)
Supplement: Supplementary file 1 [file ja5c06971_si_001.pdf]

SUPPORTING INFORMATION

The Structure of Human IAPP Fibrils Reflects Membrane and pH Conditions

*Venus Singh Mithu[1], Karin Giller[1], Evgeny Nimerovsky[1], Kerstin Overkamp[1], Loren Andreas[1], Stefan Becker[1], Christian Griesinger[1]**

[1]Department of NMR-based Structural Biology, Max Planck Institute of Multidisciplinary Sciences, Göttingen 37077, Germany

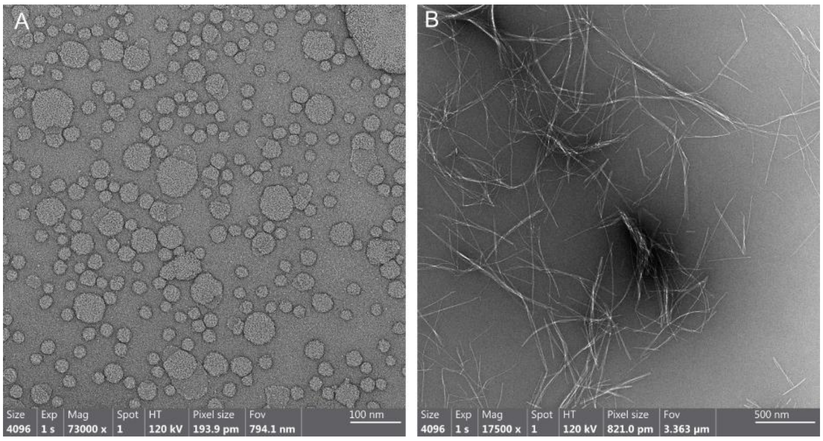

Figure S1: Negative-stain electron micrograph of (A) small unilamellar vesicles of POPC:POPS (8:2) phospholipids and (B) fibrils formed by 20μM hIAPP (without lipids) in 10mM phosphate buffer (pH 7.4) containing 50mM NaCl, incubated for 66 hours at 37°C.

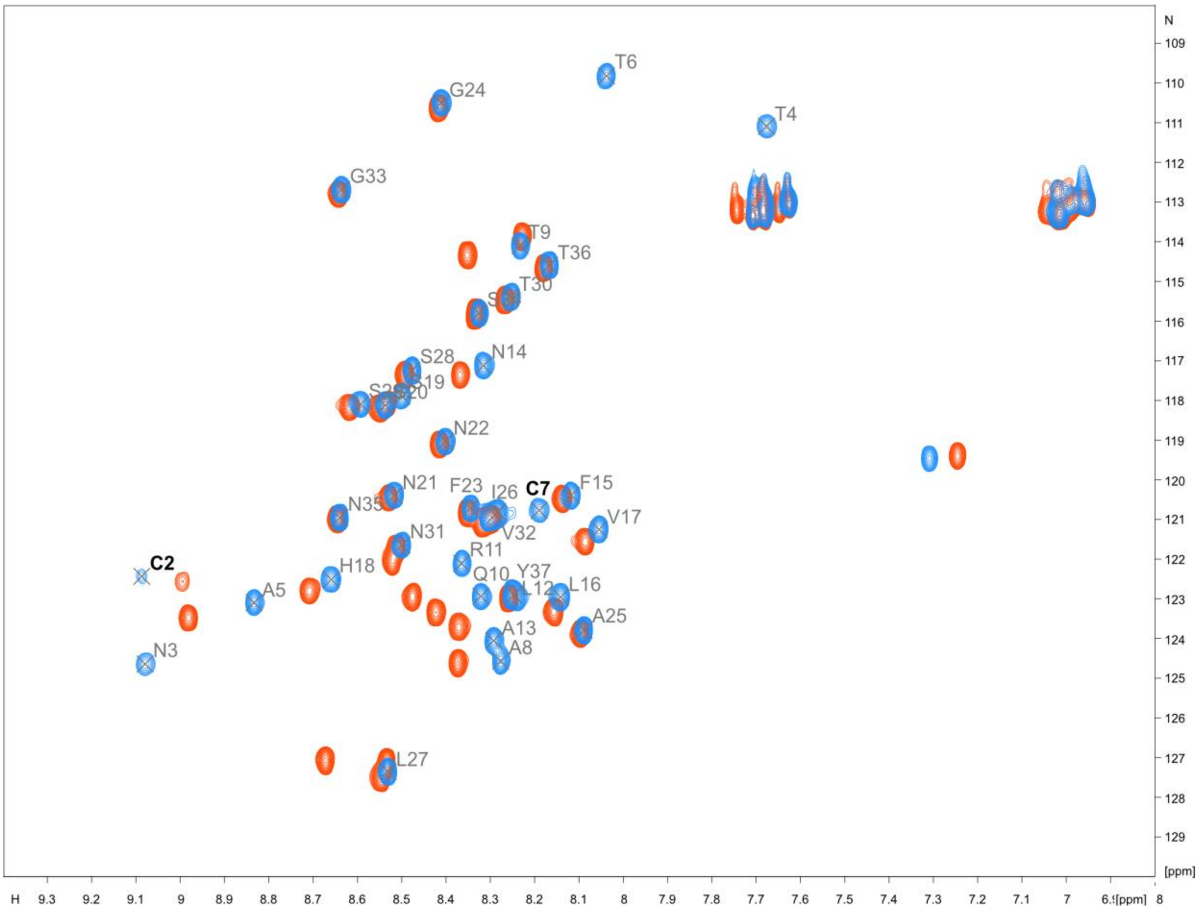

Figure S2: ^{15}N-^{1}H HSQC spectra of 75 μM hIAPP with (Oxidized, blue cross-peaks) and without (Reduced, red cross-peaks) C2-C7 disulfide bond. Spectra were acquired in 30 mM acetate buffer (pH 5.3, No NaCl) at 4°C.

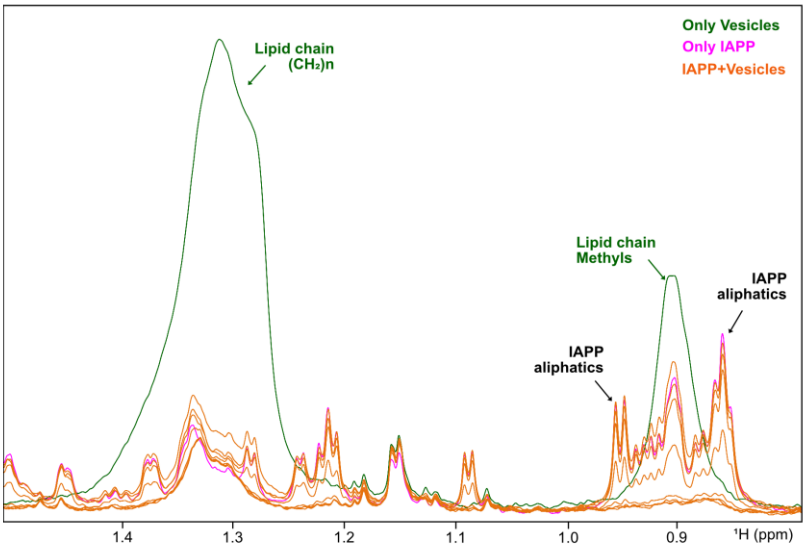

Figure S3: Overlay of 1D ^{1}H NMR spectra showing 20 µM hIAPP alone (pink) and time-dependent spectra of 20 µM hIAPP with 100 µM POPC:POPS (8:2) vesicles (orange), demonstrating a progressive loss of signal intensity consistent with peptide aggregation. Lipid signals from pure vesicles (green, 100 µM POPC:POPS (8:2)) are rapidly attenuated upon peptide addition, indicating immediate membrane interaction.

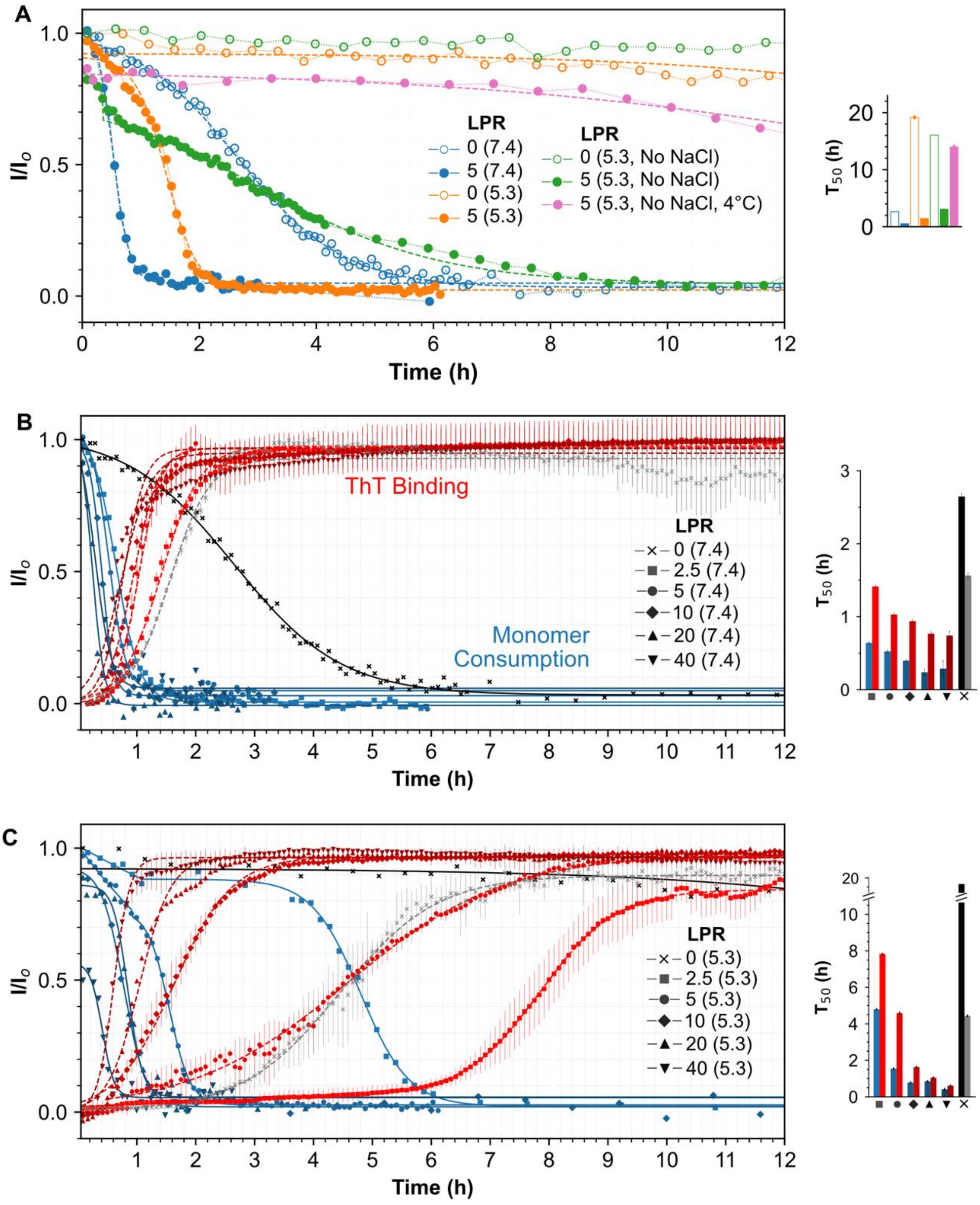

Figure S4: (A) Time-dependent decay of normalized NMR peak intensity of soluble hIAPP monomers at different lipid-to-peptide ratios (LPRs) and buffer conditions (see legend). NMR-derived monomer consumption curves (blue) and normalized ThT fluorescence intensities (red) for various LPRs (see legend) (B) in pH 7.4 buffer containing 50 mM NaCl and (C) in pH 5.3 buffer containing 50 mM NaCl. In the absence of lipids, the ThT T_{50} is lower than the monomer T_{50}, indicating that β-sheet-rich species form while monomer signals remain detectable. This contrasts with the lipidic case, where ThT fluorescence

consistently lags monomer consumption. The persistence of NMR signal suggests that flexible regions may still be visible, even after partial folding.

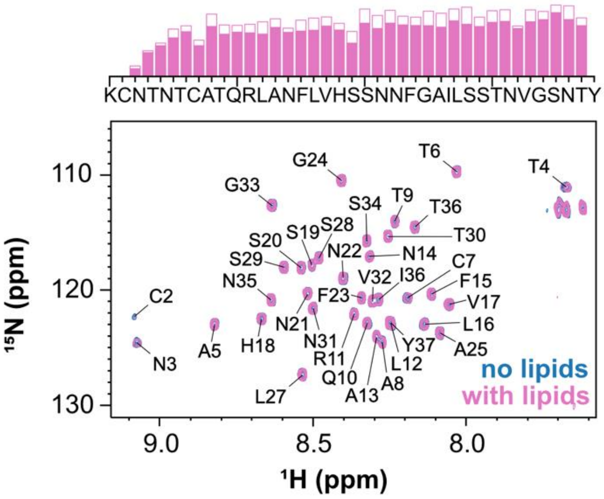

Figure S5: Overlay of ^{15}N-^{1}H HSQC spectra of 20 µM hIAPP in the absence and presence of 100 µM POPC:POPS (8:2) vesicles. Spectra were acquired in 30 mM acetate buffer (pH 5.3, No NaCl) at 4°C. The upper panel displays the relative reduction in peak intensities (pink-filled bars) upon vesicle interaction, reflecting residue-specific perturbations due to membrane binding. Peak assignment is adopted from Camargo et al.[1]

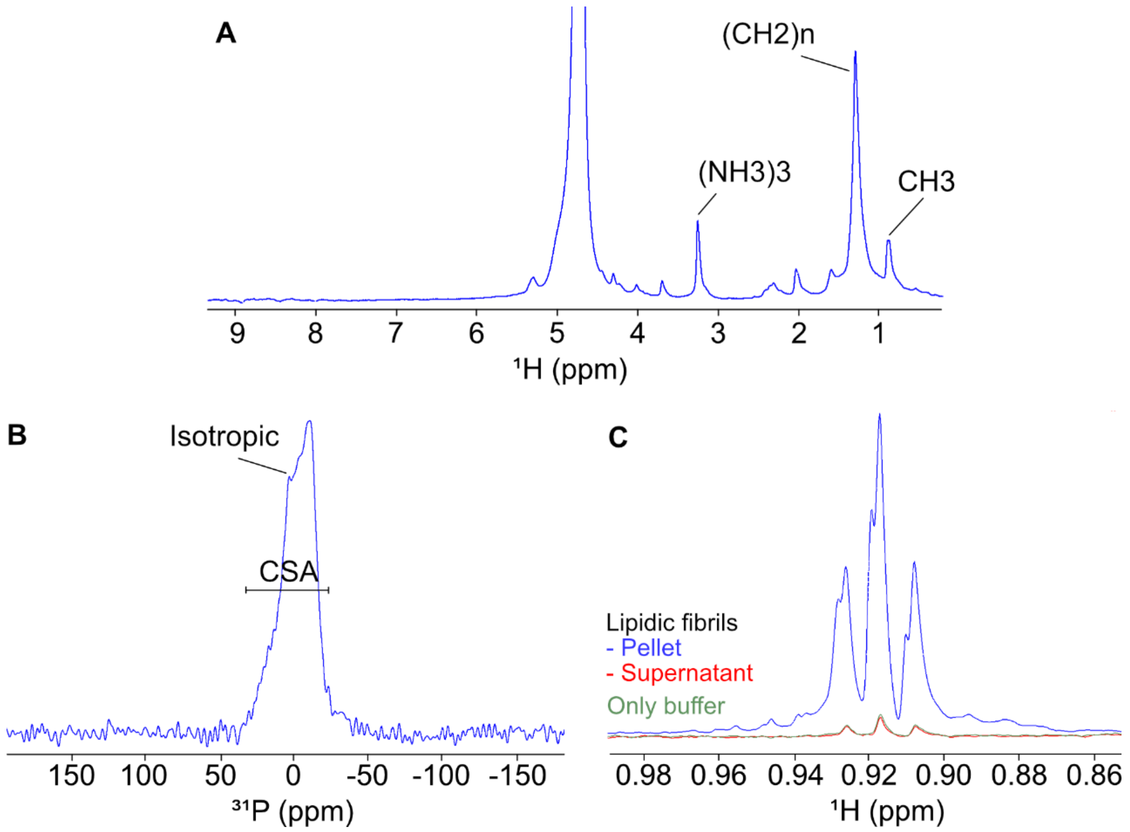

Figure S6. (A) ^{1}H 1D solid-state NMR spectrum of lipidic IAPP fibrils prepared in phosphate buffer (pH 7.4, 50 mM NaCl), recorded at 800 MHz with 55 kHz MAS. Key lipid peaks are labeled. (B) ^{31}P solid-state NMR spectrum of the same fibrils under static conditions, showing the characteristic CSA pattern and the isotropic peak. (C) (A) ^{1}H 1D NMR spectrum of lipidic IAPP fibrils prepared in phosphate buffer (pH 7.4, 50 mM NaCl), recorded at 800 MHz with 55 kHz MAS. Key lipid peaks are labeled. (B) ^{31}P solid-state NMR spectrum of the same fibrils under static conditions, showing the characteristic CSA pattern and a minor isotropic peak. (C) Overlay of ^{1}H 1D spectra in methanol-d_4 highlighting the methyl (–CH_3) region from lipid side chains in the fibril pellet (blue), supernatant (10% v/v, red), and phosphate buffer control (10% v/v, green).

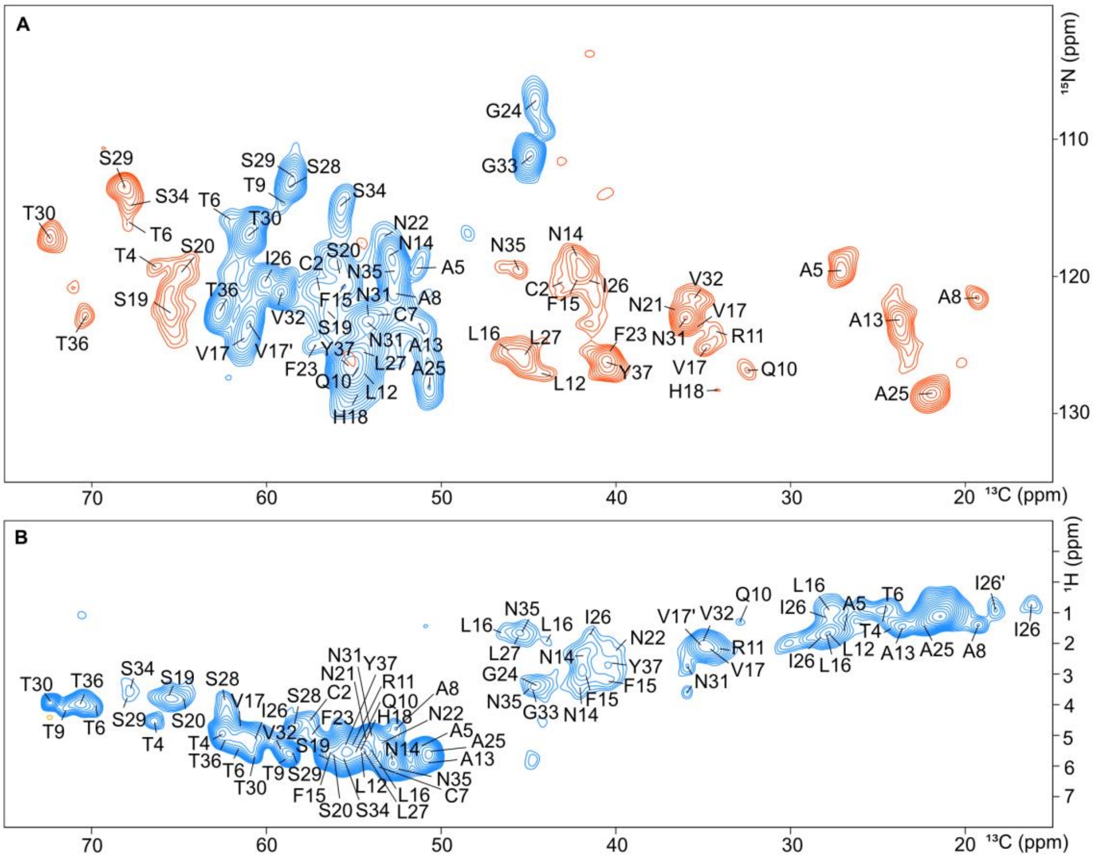

Figure S7: (A) 2D ^{15}N-^{13}C skyline projection of 3D hCANH (blue) and 3D hCBcaNH (red) spectra of pH 7.4 lipidic fibrils. (B) 2D hCH spectrum of pH 7.4 lipidic fibrils.

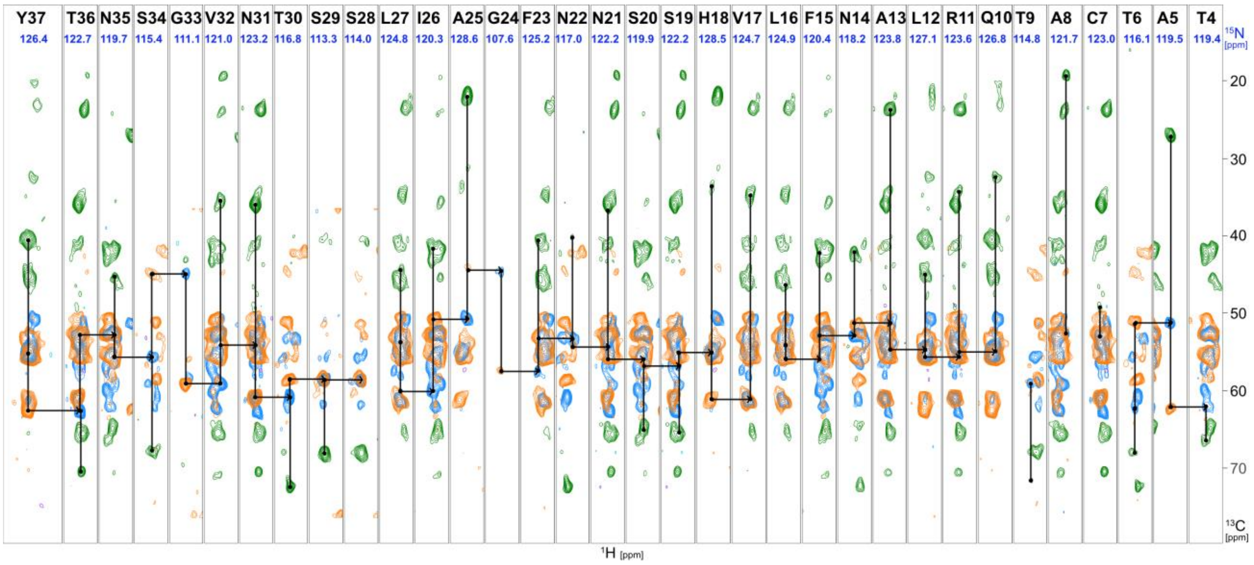

Figure S8: Sequential walk along hIAPP shown by an overlay of 3D hCANH (blue), hCAcoNH (orange), and hCBcaNH (green) spectra at selected ^{15}N frequencies, corresponding to assigned residues in pH 7.4 lipidic fibrils.

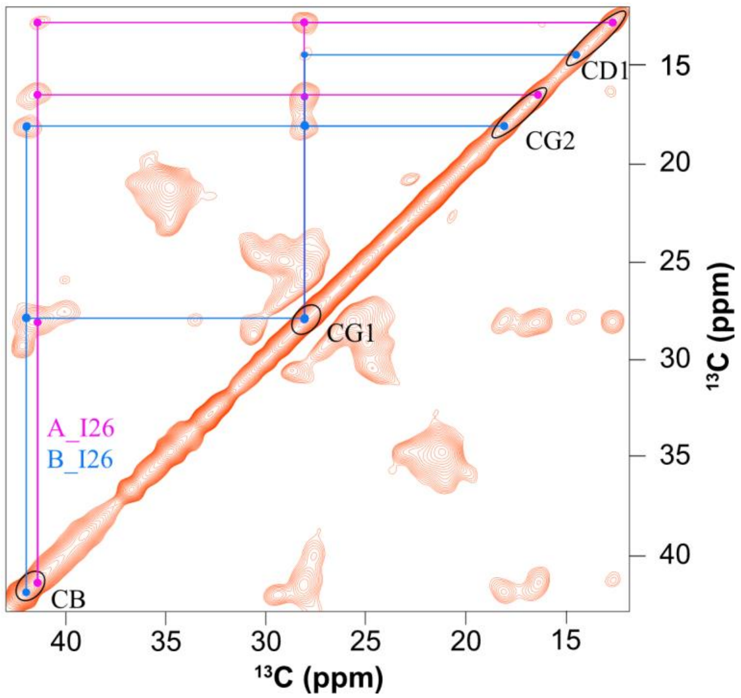

Figure S9: A selected region of the 2D hCC spectrum with 100ms DARR mixing. Intra-residue cross-peaks corresponding to two distinct conformers of I26 in hIAPP lipidic fibrils are highlighted.

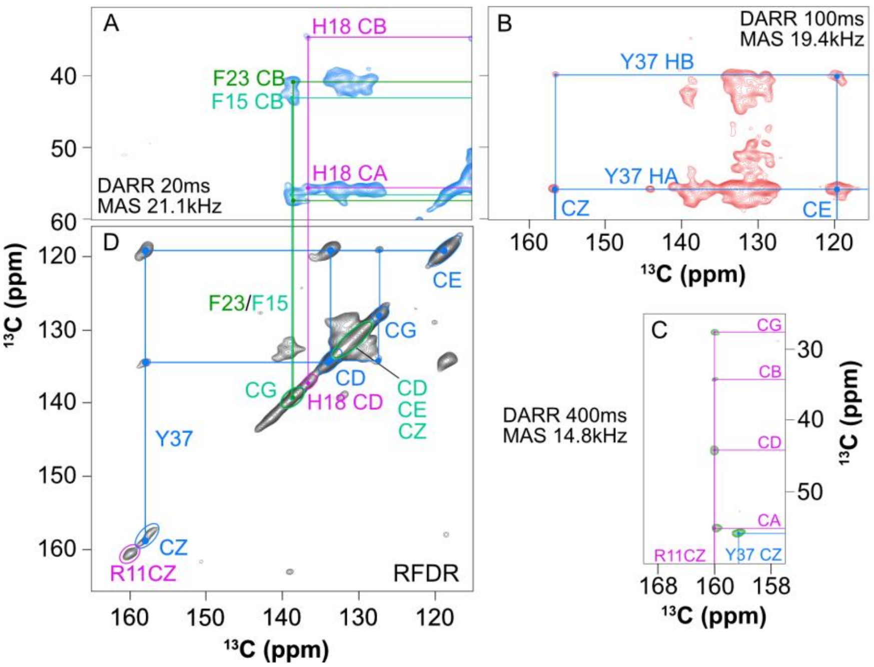

Figure S10: Selective regions of 2D hCC DARR spectra recorded under different MAS conditions with variable mixing times, highlighting intra-residue cross-peaks corresponding to R11, F15, H18, F23, and Y37 in pH 7.4 lipidic fibrils.

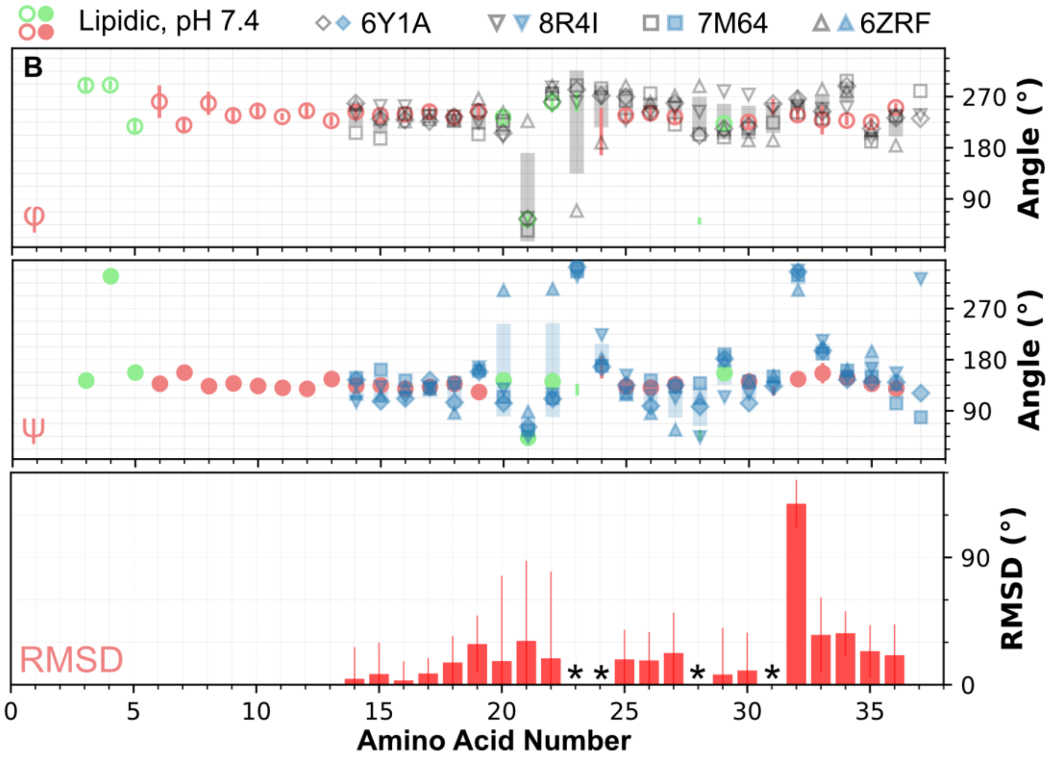

Figure S11: TALOS-N predicted φ (open circles, top panel) and ψ (filled circles, middle panel) dihedral angles (360° scale) and associated errors for pH 7.4 lipidic fibrils, compared to residues 14–36 in various S-type cryo-EM structures (see legend). Cryo-EM angle distributions are shown as shaded bars representing the error range around the average angle from four structures per residue. The bottom panel shows root-mean-square deviation (RMSD) of torsional angles between pH 7.4 fibrils and the averaged cryo-EM S-type structures. Residues with low-quality predictions are excluded and marked with asterisks. An enlarged version of panel B is available as Figure S11.

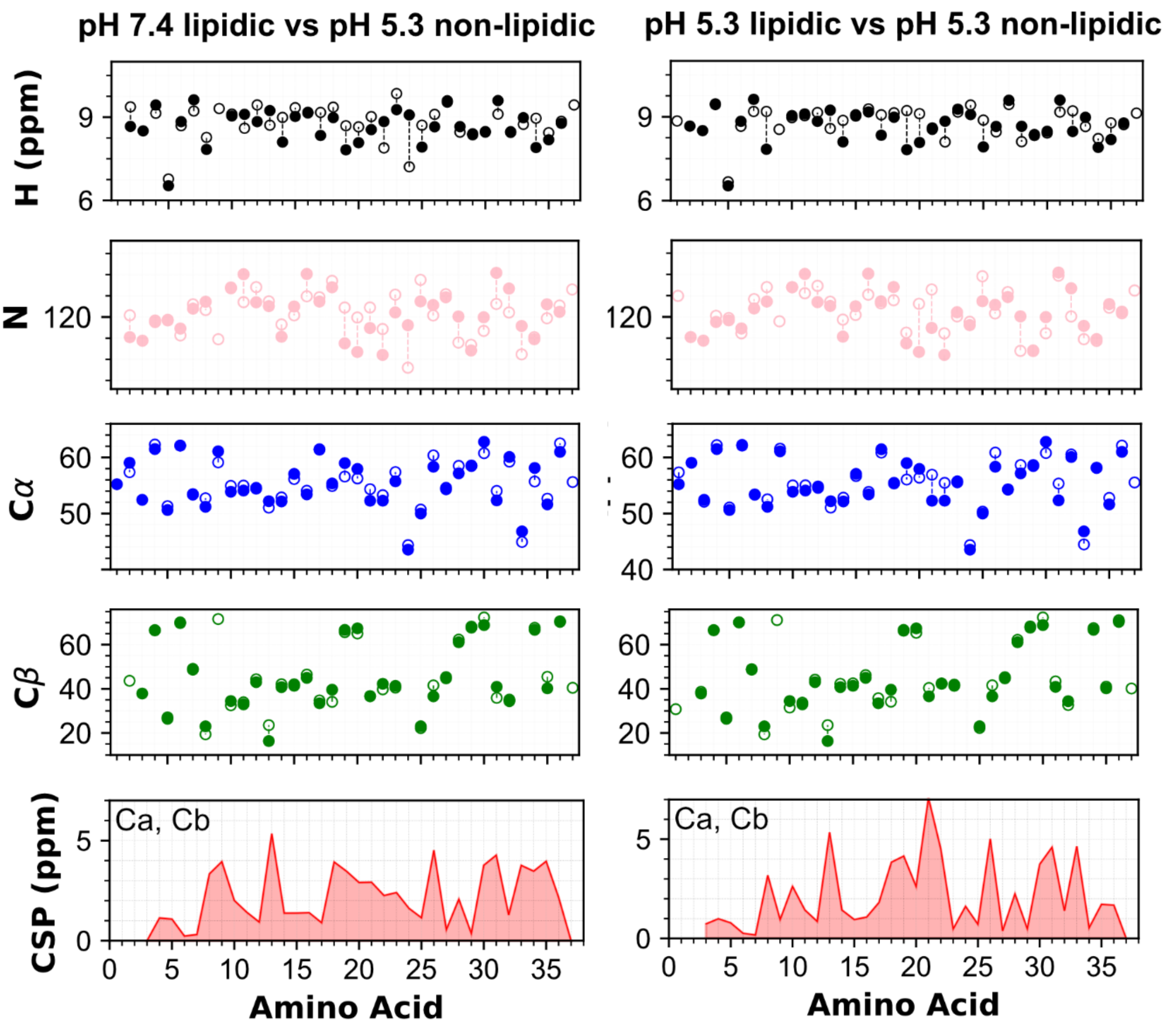

Figure S12: Comparison of chemical shifts between pH 7.4 lipidic fibrils (open circles) and non-lipidic fibrils reported by Suladze et al. (filled circles). Chemical shift perturbations (CSPs) were calculated using Cα and Cβ values. Notable chemical shift differences are observed for residues A8, Q9, A13, H18, S19, S20, I26, and much of the C-terminal region (30–36), indicating structural divergence between lipidic fibrils at pH 7.4 and non-lipidic fibrils at pH 5.3. These differences are expected given the distinct aggregation conditions—pH, agitation, and lipid presence. Importantly, even lipidic fibrils formed at pH 5.3 show significant CSP relative to non-lipidic fibrils, underscoring the strong structural influence of lipids.

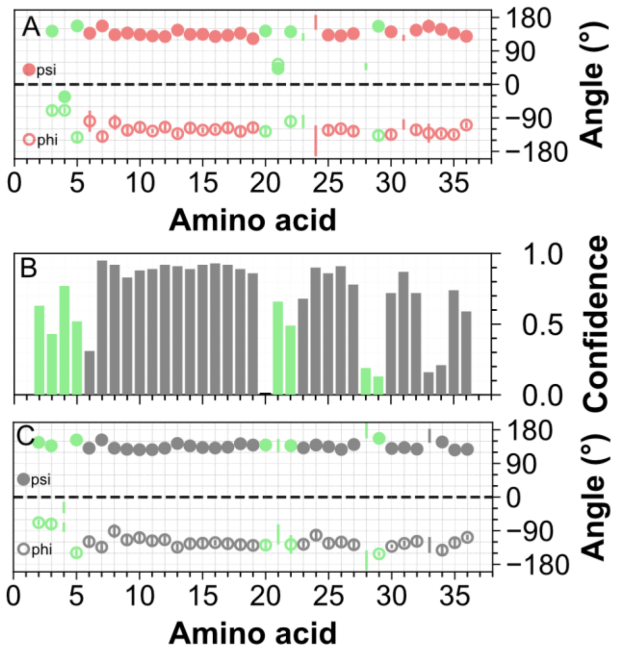

Figure S13: (A) TALOS-N predicted φ (open cirles) and ψ (filled circles) dihedral angles for pH 7.4 lipidic fibrils. (B) TALOS-N prediction confidence for secondary structure elements in pH 5.3 lipidic fibrils. C) TALOS-N predicted φ (open cirles) and ψ (filled circles) dihedral angles for pH 5.3 lipidic fibrils.

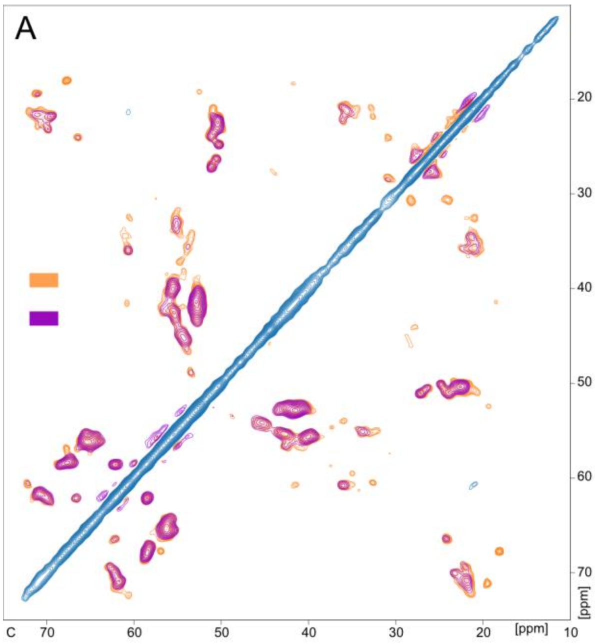

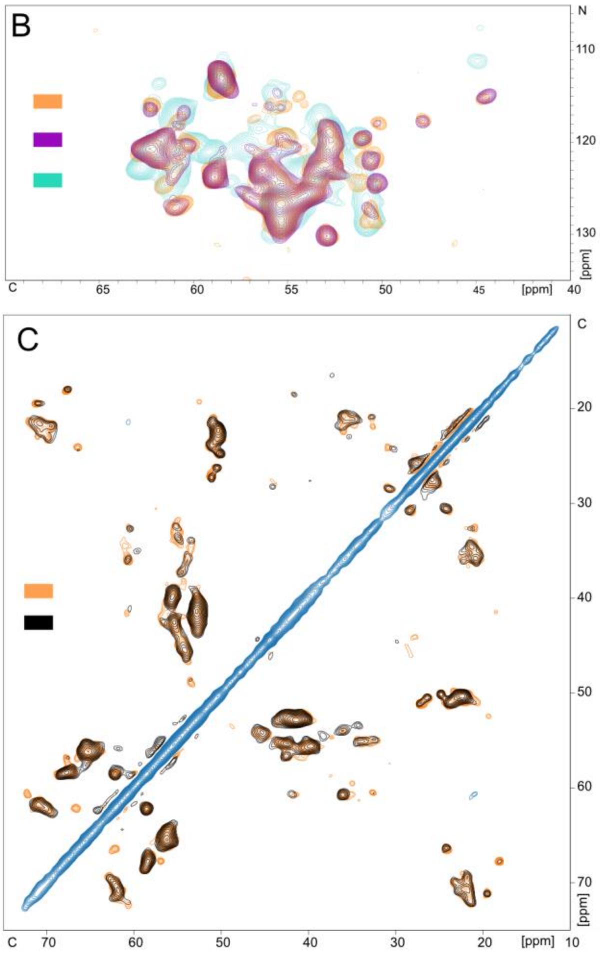

	Fibril growth conditions	NMR data recording conditions
(orange)	pH 5.3 (50mM NaCl)	pH 5.3 (50mM NaCl)
(purple)	pH 5.3 (50mM NaCl)	pH 7.4 (50mM NaCl)
(black)	pH 5.3 (No NaCl)	pH 5.3 (No NaCl)
(cyan)	pH 7.4 (50mM NaCl)	pH 7.4 (50mM NaCl)

Figure S14: Overlay of (A, C) 2D hCC SPEPS spectra and (B) 2D C–N projections from 3D hCaNH spectra of lipidic fibrils grown and measured under buffer conditions listed in the accompanying table. Panels A and B show that buffer exchange of preformed pH 5.3 fibrils into pH 7.4 phosphate buffer results in minimal spectral changes, with the spectra (purple and orange cross-peaks) remaining distinct from those of fibrils grown directly in pH 7.4 phosphate buffer (cyan). Panel C highlights the close spectral similarity between fibrils grown at pH 5.3 with (orange) and without (black) salt, suggesting negligible structural variation. In the C–C spectra, cross-peak color coding in the table refers to off-diagonal peaks only; diagonal contours are shown in blue.

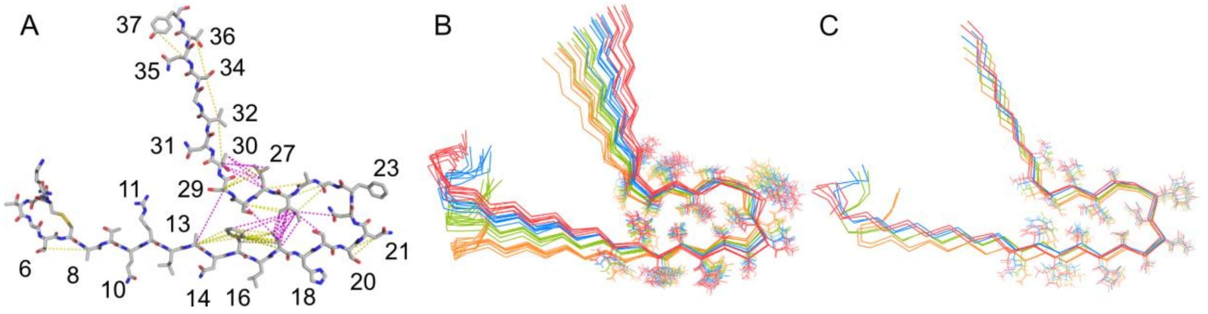

Figure S15: (A) Long-range (|i–j| > 2, magenta) and short-range (|i–j| = 2, yellow) inter-residue contacts identified from 2D and 3D spectra via H–H and C–C through-space magnetization transfers, mapped onto the protofilament structure of hIAPP lipidic fibrils at pH 7.4. (B, C) Ten lowest-energy conformers generated from CYANA calculations, each consisting of four protofilaments arranged in a cross-β architecture. Individual monomers are shown in different colors for clarity. Most conformers exhibit a subtle β-sheet twist (B), while two display a flatter β-sheet arrangement (C).

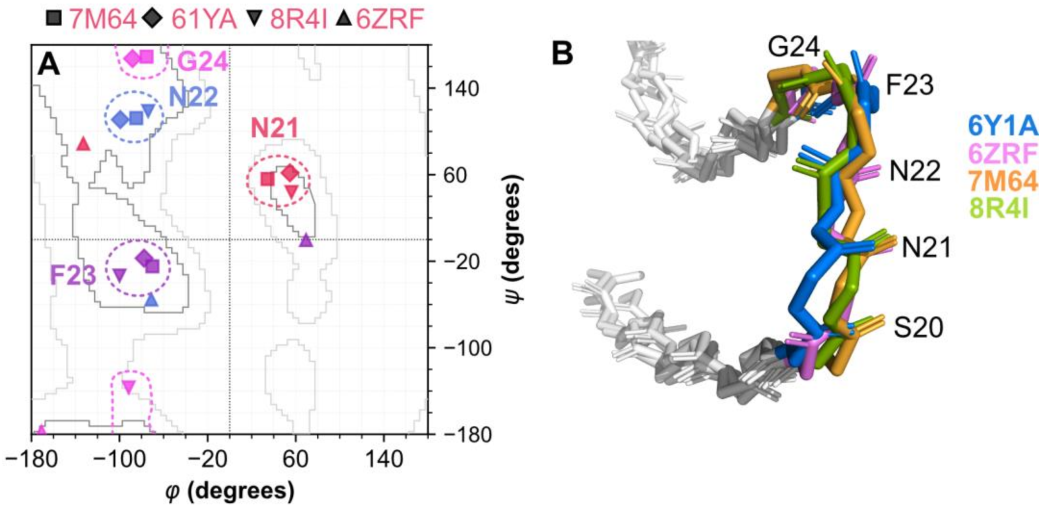

Figure S16: (A) Dihedral angles of loop region residues extracted from four distinct S-type non-lipidic hIAPP fibril structures. Structures with similar dihedral angles are grouped with dotted loops. (B) Close-up view of the backbone atom arrangement for these residues in aligned PDB structures, highlighting the distinct backbone orientations in 6ZRF.

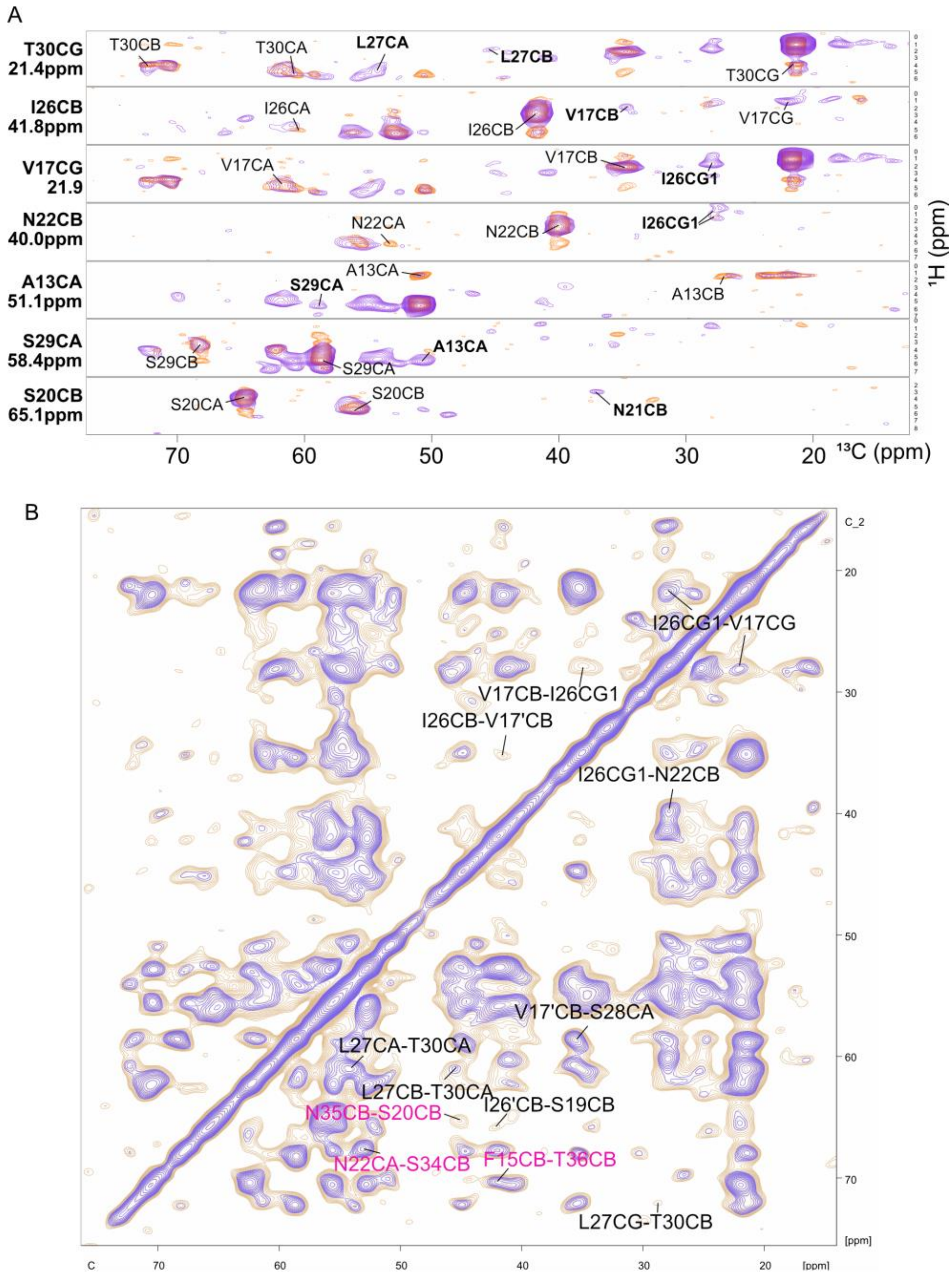

Figure S17: (A) Selective 2D planes corresponding to specific carbon chemical shifts (indicated on the left), extracted from the 3D hCCH GODIST (orange) and 3D hChhCH MODIST (purple) spectra. Bold peaks mark inter-residue cross-peaks involving the indicated carbon in the MODIST spectrum. (B) 2D hCC DARR spectrum of lipidic fibrils at pH 7.4, recorded with a 300 ms mixing time. Two contour colors represent the same spectrum processed with different parameters to enhance feature visibility. Key inter-residue cross-peaks within the same protofilament are labeled in black, while potential (ambiguous) inter-protofilament contacts are indicated in magenta as follows (1) F15CB/I26CB/N14CB—T36CB, (2) N22CA/N14CA/A8CA/C7CA/N35CA—S34CB, and (3) N35CB/A27CB/G33CA — S20CB/S19CB.

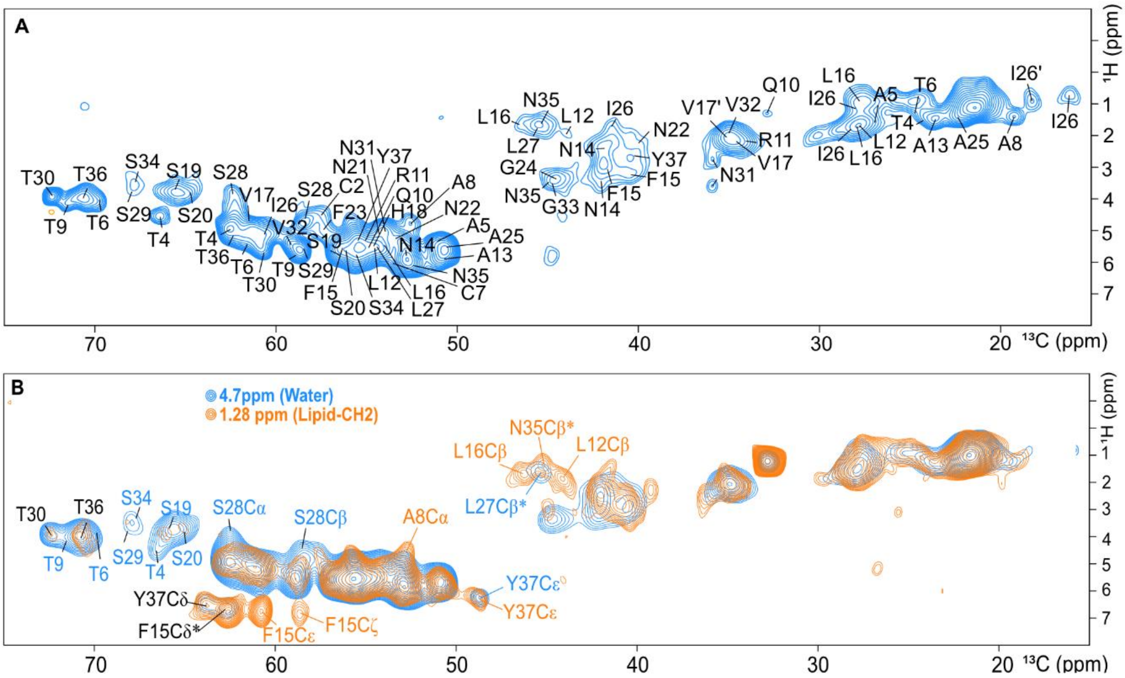

Figure S18 (A) (A) 2D hCH spectrum of hIAPP fibrils formed at pH 7.4 in the presence of lipids. (B) 2D plane from a 3D lipid-edited HhCH (z-mixing = 50 ms, T_2 filter = 140 Hz) spectrum showing projections corresponding to water (blue contours) and lipid methylene (orange contours) signals. Water–protein and lipid–protein contacts are labeled using the same color scheme. Black labels indicate peaks observed in both cases. Unambiguous assignments are marked with an asterisk (*). Notably, side-chain signals of F15 (aliased onto the displayed region) exhibit strong lipid interactions. This is further supported by lipid-edited peaks from L16 Cβ, L12 Cβ, and A8 Cα, suggesting close proximity of this region to the lipid environment. These contacts may contribute to the stability of the extended β-sheet in the N-terminal region of the lipidic fibrils. In contrast, weaker signals from the Cβ atoms of T4, T6, and T9 indicate that the extreme N-terminus—predicted to be unstructured by TALOS-N—remains more water-exposed. Additional signals from S28 and S29, along with L27 Cβ, S29 Cβ, and S34 Cβ, suggest water-accessibility and possible hydrophilic interactions in this region.

<u>CYANA structure calculations with ambiguous TALOS-N predictions for F23 and G24</u>

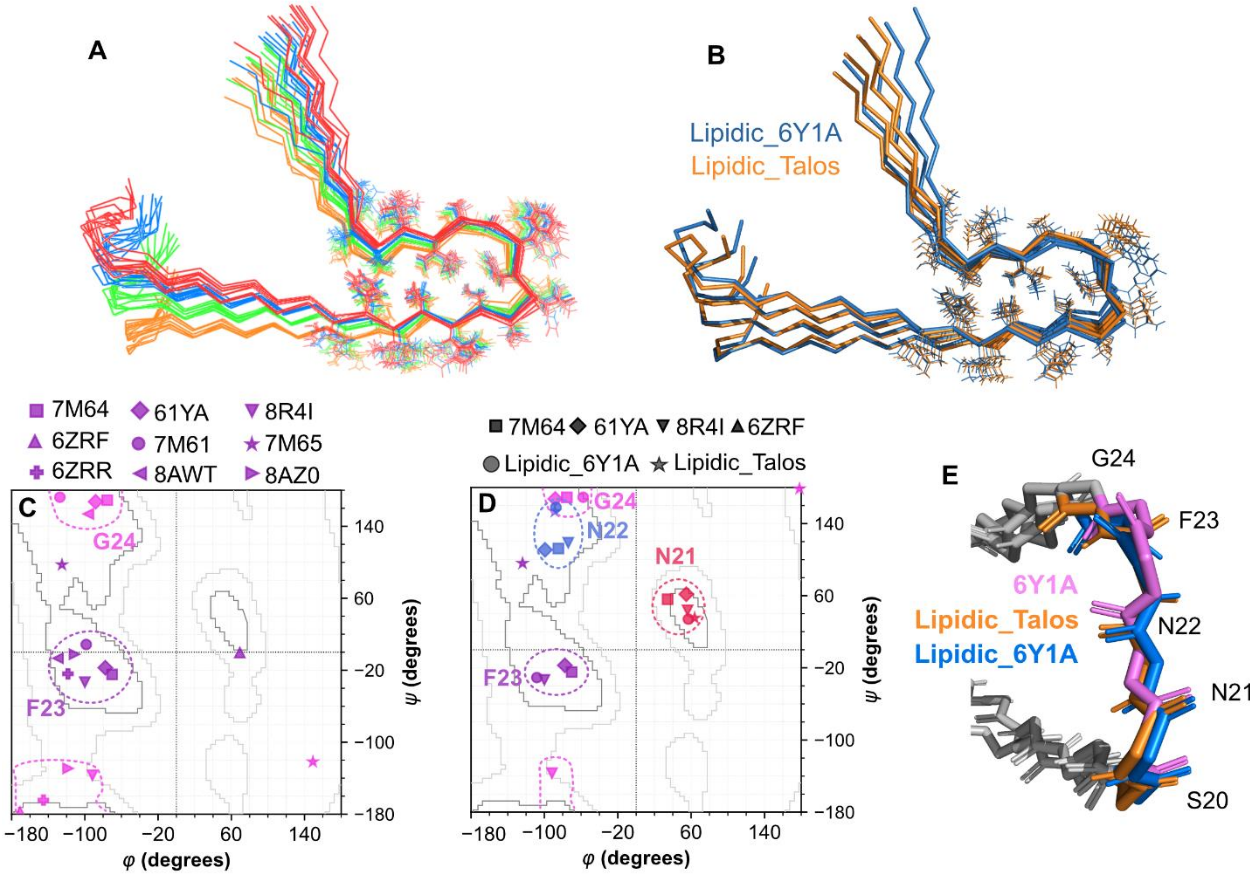

Figure S19 (A) Ensemble of the ten lowest-energy conformers generated from CYANA calculations using ambiguous dihedral angle restraints for F23 and G24. Each model comprises four protofilaments arranged in a cross-β architecture; monomers are shown in different colors for clarity. (B) Structural overlay of the lowest-energy conformer obtained using ambiguous angles (TALOS) and the one calculated using dihedral angles for F23 and G24 adopted from the 6Y1A structure, as indicated in the legend. (C) Distribution of dihedral angles for F23 and G24 across different non-lipidic fibril structures. Structures with similar values are grouped with dotted loops. (D) Comparison of dihedral angles within the loop spanning residues 21–24 in ensembles calculated using ambiguous versus 6Y1A-derived restraints. (E) Close-up view of the backbone arrangement for residues 21–24 in aligned PDB structures, highlighting the distinct backbone orientation adopted by F23 and G24 in the lipidic fibril structure calculated using ambiguous angles.

The TALOS-N predictions for residues F23 and G24 yielded ambiguous results and were therefore not recommended for direct use in structure calculations. Nonetheless, we initially performed structure calculations using these ambiguous angle restraints. **Figure S17A** shows the ten lowest-energy conformers obtained using the ambiguous angles. **Figure S17B**

compares the lowest-energy conformer with the structure calculated using dihedral angles for F23 and G24 taken from 6Y1A.

However, when comparing these angles with those in core-fold 1, we found that the increased RMSD primarily arises from the loop region, particularly residues F23 and G24. To highlight the heterogeneity in these angles, we compared the loop conformations across four S-type structures (6Y1A, 6ZRF, 7M64, and 8R4I; see **Figure S16**). As discussed in the main text, despite all adopting the same overall fold and conserved side chain orientations, the dihedral angles for F23 and G24 in 6ZRF differ significantly from the others.

With the exception of outliers like 6ZRF, most structures with core-fold 1 (**Figure 4E**) adopt similar dihedral angles for F23 and G24 (see **Figure S17C**). These differences likely reflect the resolution limits of cryo-EM reconstructions (typically between 3.0 and 4.2 Å), which can introduce small variations in loop conformations while preserving the β-arch that connects β-strands 1 and 2 in S-type structures. Therefore, we chose to adopt dihedral angles for these two residues from 6Y1A.

Figure **S17D** shows the dihedral angles in the loop region from calculations using ambiguous restraints versus those using 6Y1A-derived restraints. As expected, F23 and G24 exhibit different angles in the two ensembles, as illustrated by the backbone orientation in Figure **S17E**. Nevertheless, the side chain orientations remain conserved, consistent with all known core-fold structures.

Table S1A: Acquisition parameters for NMR measurements on pH 7.4 lipidic fibrils for sequential assignment.

	**hNH**	**hCH**	**hCANH**	**hCAcoNH**	**hCOcaNH**	**hCONH**	**hCBcaNH**	**hCBcacoNH**
**H 90° (μs)**	2.7	2.3	2.7	2.7	2.7	2.7	2.7	2.7
**N 90° (μs)**	4.7	4.7	4.7	4.7	4.7	4.7	4.7	4.7
**C 90° (μs)**	3.6	3.6	3.6	3.6	3.6	3.6	3.6	3.6
**H→N (kHz)**<br>**CP (μs)**	121→45<br>800							
**N→H (kHz)**<br>**CP (μs)**	45→115<br>700		45→117<br>1100	45→117<br>1100	45→117<br>800	45→117<br>1100	45→117<br>800	45→119<br>1000
**H→C (kHz)**<br>**CP (μs)**		117→46<br>500	120→48<br>700	120→48<br>700	119→48<br>3600	119→48<br>3600	119→48<br>3600	122→48<br>3600
**C→H (kHz)**<br>**CP (μs)**		48→113<br>400						
**C→N (kHz)**<br>**SPEPS (ms)**			13→41<br>2.91	13→41<br>2.91	13→42<br>2.91	13→42<br>2.91	13→41<br>2.91	13→41<br>2.91
**C→C (kHz)**<br>**hSPEPS (ms)**				55.6	55.6		1.25×MAS	1.25×MAS
**SW (H) (ppm)**	49.6	49.6	49.6	49.6	49.6	49.6	49.6	49.6
**SW (N) (ppm)**	200		40	40	40	40	40	40
**SW (C) (ppm)**		40	40	40	30	30	80	80
**AQ (H) (ms)**	12.9	12.9	12.9	12.9	12.9	12.9	12.9	12.9
**AQ (N) (ms)**	15.8	6.2	6.2	6.2	6.2	6.2	6.2	6.2
**AQ (C) (ms)**		4.5	4.5	4.5	4.6	4.6	4.0	4.0
**D1 (s)**	0.3	0.3	0.3	0.3	0.3	0.3	0.3	0.3
**MAS (kHz)**	55.0	55.0	55.0	55.0	55.0	55.0	55.0	55.0

Table S1B: Acquisition parameters for NMR measurements on pH 7.4 lipidic fibrils for sequential assignment and long-range contacts.

	**hCC**<br>SPEPS	**hCC**<br>RFDR	**hCC**<br>DARR	**hCCH**<br>GODIST	**hChhCH**<br>MODIST	**hCC**<br>DARR	**hCC**<br>DARR	**hCC**<br>DARR
**H 90° (μs)**	2.7	2.8	2.3	2.4	2.4	2.7	2.7	2.6
**N 90° (μs)**	4.7	3.7	3.7	3.5	3.5			
**C 90° (μs)**	3.6	3.0	3.15	3.2	3.2	3.2	3.6	3.6
**H→C (kHz)**<br>**CP (μs)**	124→48<br>900	121→48<br>500	88→39<br>400	123→48<br>700	108→41<br>350	72→58<br>700	76→60<br>1100	85→60<br>1500
**C→H (kHz)**<br>**CP (μs)**				119→48<br>500	106→41<br>250			
**C→C (kHz)**<br>**Mixing time (ms)**	1.16	4.65	1.0×MAS<br>300	0.5×MAS<br>5.81	0.25×MAS<br>5.81	1.0×MAS<br>10, 20, 100, 200, and 400		
**SW (H) (ppm)**			49.6	49.6	49.6			
**SW (C) (ppm)**	327.2	327.2	295.9	70.0	70.0	298.9	348.7	249.1
**AQ (H) (ms)**				12.9	12.9			
**AQ (C) (ms)**	7.8	7.8	8.6	6.0	4.0	8.1	6.1	5.4
**H Decoupling (SwTPPM) (kHz)**	13.75	13.75	90.0	13.75	13.75	79.0	80.0	80.1
**D1 (s)**	0.3	0.3	2.0	0.3	0.3	2.0	2.0	2.0
**MAS (kHz)**	55.0	55.0	14.987	55.0	55.0	14.987	19.444	21.111
**Rotor (mm)**	1.3	1.3	1.9	1.3	1.3	1.9	1.9	1.9
**Field (T)**	18.8	18.8	22.3	18.8	18.8	22.3	22.3	22.3

Table S1C: Acquisition parameters for NMR measurements on pH 5.3 lipidic fibrils for sequential assignment.

	**hNH**	**hCH**	**hCANH**	**hCAcoNH**	**hCOcaNH**	**hCBcaNH**
**H 90° (μs)**	2.3	2.3	2.4	2.4	2.4	2.4
**N 90° (μs)**	3.8	3.8	3.8	3.8	3.8	3.8
**C 90° (μs)**	3.2	3.2	3.2	3.2	3.2	3.2
**H→N (kHz)**<br>**CP (μs)**	109→37<br>500					
**N→H (kHz)**<br>**CP (μs)**	37→101<br>800		37→97<br>600	37→97<br>600	37→97<br>600	37→97<br>600
**H→C (kHz)**<br>**CP (μs)**		107→48<br>400	105→45<br>500	105→45<br>500	105→45<br>500	105→45<br>500
**C→H (kHz)**<br>**CP (μs)**		49→103<br>400				
**C→N (kHz)**<br>**SPEPS (ms)**			12→40<br>2.91	12→40<br>2.91	12→40<br>2.91	12→40<br>2.91
**C→C (kHz)**<br>**hSPEPS (ms)**				51.7<br>1.16	51.8<br>1.16	1.25×MAS<br>1.16
**SW (H) (ppm)**	49.6	49.6	49.6	49.6	49.6	49.6
**SW (N) (ppm)**	200		40	40	40	40
**SW (C) (ppm)**		120	40	40	30	80
**AQ (H) (ms)**	12.9	12.9	12.9	12.9	12.9	12.9
**AQ (N) (ms)**	15.8	6.2	6.2	6.2	6.2	6.2
**AQ (C) (ms)**		10.6	4.5	4.5	4.6	4.0
**D1 (s)**	0.3	0.3	0.3	0.3	0.3	0.3
**MAS (kHz)**	55.0	55.0	55.0	55.0	55.0	55.0

Table S1D: Acquisition parameters for NMR measurements on pH 7.4 lipidic fibrils for sequential assignment and long-range contacts.

	**hCC**<br>SPEPS	**hCC**<br>RFDR	**hCCH**<br>GODIST	**hChhCH**<br>MODIST
**H 90° (μs)**	2.3	2.4	2.3	2.3
**N 90° (μs)**	3.8	3.3	3.8	3.8
**C 90° (μs)**	3.2	3.2	3.2	3.2
**H→C (kHz)**<br>**CP (μs)**	105→45<br>400	105→45<br>400	108→46<br>500	108→45<br>250
**C→H (kHz)**<br>**CP (μs)**			46→108<br>300	45→110<br>260
**C→C (kHz)**<br>**Mixing time (ms)**	68.8<br>1.16	78.1<br>4.65	0.5×MAS<br>10.5	0.25×MAS<br>4.65
**SW (H) (ppm)**			49.6	49.6
**SW (C) (ppm)**	327.2	327.2	70.0	70.0
**AQ (H) (ms)**			12.9	12.9
**AQ (C) (ms)**	7.8	7.8	6.0	4.0
**D1 (s)**	0.3	0.3	0.3	0.3
**MAS (kHz)**	55.0	55.0	55.0	55.0

Table S2A: Chemical shifts in pH 7.4 lipidic fibrils used for TALOS-N-based prediction of backbone torsion angles and secondary structure propensities.

AAn	AA	Co	Cα	Cβ	^{N}H	Hα	N_H
1	LYS	-	-	-	-	-	-
2	CYS	173.17	57.37	43.63	9.37	4.62	120.41
3	ASN	-	-	-	-	-	-
4	THR	173.98	62.30	66.47	9.14	4.93	119.18
5	ALA	176.39	51.31	27.08	6.77	5.46	119.34
6	THR	174.21	62.12	69.78	8.69	5.42	115.66
7	CYS	172.06	53.46	49.06	9.22	6.07	123.00
8	ALA	176.54	52.72	19.39	8.27	5.03	121.66
9	THR	173.73	59.12	71.61	9.31	5.76	114.75
10	GLN	0.00	54.93	32.54	9.11	5.52	126.79
11	ARG	0.00	54.98	33.91	8.60	5.32	123.52
12	LEU	175.11	54.43	44.33	9.44	5.60	127.06
13	ALA	176.67	51.04	23.54	8.71	5.80	123.70
14	ASN	174.37	52.87	42.09	8.99	5.88	118.25
15	PHE	173.51	56.15	42.06	9.34	5.64	120.40
16	LEU	175.29	54.06	46.46	9.15	5.55	124.90
17	VAL	174.16	61.39	34.74	9.17	4.63	124.67
	VAL′	175.00	60.90	35.53	9.14	5.18	123.98
18	HIS	-	54.90	34.11	9.36	5.41	128.54
19	SER	174.01	56.59	65.66	8.69	5.68	122.24
20	SER	173.98	56.25	65.07	8.65	5.61	119.89
21	ASN	173.51	54.34	36.73	9.02	5.13	122.25
22	ASN	173.53	53.27	39.76	7.89	5.23	117.16
23	PHE	174.96	57.39	40.54	9.85	4.99	125.28
24	GLY	172.59	44.36	-	7.21	3.31	108.03
25	ALA	174.77	50.70	22.20	8.71	5.56	128.78
26	ILE	174.16	60.37	41.55	9.10	5.12	120.38
	ILE′	173.63	60.56	41.77	-	-	-
27	LEU	175.66	54.58	45.31	9.54	5.53	125.39
28	SER	174.05	58.50	62.3	8.46	4.43	113.97
29	SER	171.98	58.57	68.19	8.40	5.57	113.45
30	THR	174.24	60.76	72.34	8.46	5.60	116.73
31	ASN	172.48	54.03	35.90	9.11	5.11	123.09
32	VAL	174.58	59.25	35.12	8.45	5.42	121.07
33	GLY	171.50	44.92	-	8.74	3.43	111.19
34	SER	172.51	55.71	67.65	8.96	5.78	115.23
35	ASN	176.28	52.65	45.45	8.44	6.07	119.71
36	THR	172.14	62.52	70.60	8.85	5.16	122.71
37	TYR	176.35	55.62	40.46	9.44	5.31	126.51

Table S2B: Side-chain chemical shifts in pH 7.4 lipidic fibrils.

4	THR	Cγ2 24.41, Hγ1 1.43 δ ε γ ζ
6	THR	Cγ2 24.62, Hγ1 1.21
9	THR	Cγ2 24.12
10	GLN	Cγ 34.38, Cδ 179.14
11	ARG	Cγ 27.98, Cδ 44.07, Cζ 159.88, Hγ2/3 1.53
12	LEU	Cγ 27.96, Cδ1/2 25.77, Hγ 1.73

14	ASN	Cγ 176.68
15	PHE	Cγ 138.70, Cδ1/2 132.24, Cε1/2 130.68, Cζ 128.48
16	LEU	Cγ 28.08, Cδ1/2 25.62, Hγ 1.71, Hδ1/2 0.91
17	VAL	Cγ1/2 21.79, Hγ1/2 1.22
17	VAL′	Cγ1/2 20.99, Hγ1/2 0.90
18	HIS	Cε1 136.57
21	ASN	Cγ 177.53
22	ASN	Cγ 176.24
23	PHE	Cγ 138.45, Cδ1/2 131.31
26	ILE	Cγ1 28.19, Cγ2 16.50, Cδ1 12.71, Hγ 0.86, Hδ1 0.73, Hγ12 1.77
26	ILE′	Cγ1 27.75, Cγ2 18.22, Cδ1 14.38, Hγ 0.84, Hδ1 1.00, Hγ12 1.55
27	LEU	Cγ 28.87, Cδ1/2 24.61, Hγ 1.92, Hδ1/2 1.00
30	THR	Cγ2 21.43, Hγ1 1.15
31	ASN	Cγ 178.05, Hδ21 7.43, Hδ22 6.84, Nδ2 112.38
32	VAL	Cγ1/2 21.16, Hγ1/2 0.69
36	THR	Cγ2 21.85, Hγ1 1.13
37	TYR	Cγ 127.67, Cδ 133.55, Cε 118.47, Cζ 157.66

Table S3A: Chemical shifts in pH 5.3 lipidic fibrils used for TALOS-N-based prediction of backbone torsion angles and secondary structure propensities.

AAn	AA	Co	Cα	Cβ	^{N}H	Hα	N_H
1	LYS	173.52	55.21	30.74	8.85	5.46	124.94
2	CYS	-	-	-	-	-	-
3	ASN	176.68	52.10	38.66	0.00	0.00	0.00
4	THR	174.80	62.22	66.57	9.46	4.60	120.25
5	ALA	176.18	51.03	27.03	6.67	5.28	119.72
6	THR	173.89	62.24	70.11	8.66	5.09	116.14
7	CYS	172.05	53.39	48.91	9.19	6.04	124.18
8	ALA	176.72	52.55	19.37	9.19	5.99	127.01
9	THR	173.04	61.55	71.14	8.55	5.21	118.96
10	GLN	174.71	55.01	31.52	8.97	0.00	127.00
11	ARG	174.61	55.03	33.74	9.04	0.00	125.56
12	LEU	175.11	54.83	44.16	9.15	5.50	127.30
13	ALA	176.47	51.01	23.57	8.58	5.63	123.57
14	ASN	173.60	52.77	42.41	8.87	5.56	119.36
15	PHE	173.28	56.71	42.48	9.07	5.68	120.46
16	LEU	175.21	53.94	46.21	9.28	5.64	125.24
17	VAL	174.64	60.84	35.68	9.07	4.83	123.20
	VAL′	173.81	61.14	34.66	8.91	4.76	126.65
18	HIS	174.47	55.47	34.17	9.12	5.59	123.92
19	SER	173.24	56.06	66.31	9.22	5.48	116.29
20	SER	173.42	56.37	65.53	9.11	5.86	123.08
21	ASN	173.83	56.92	40.39	8.60	4.59	126.45
22	ASN	174.89	55.48	42.39	8.10	5.75	116.13
	ASN′	173.88	52.76	40.74	8.55	5.80	119.63
23	PHE	174.96	55.60	42.07	9.17	5.64	120.13
	PHE′	173.11	55.98	40.77	9.06	5.97	124.81
24	GLY	171.84	44.41	0.00	9.30	3.37	118.84
25	ALA	175.54	50.35	22.25	8.88	5.65	129.56
26	ILE	174.82	60.84	41.56	8.46	4.95	120.82
	ILE′	173.80	58.90	37.60	8.91	5.00	123.60
27	LEU	174.69	54.26	45.20	9.44	5.59	125.83
28	SER	174.00	58.65	62.26	8.11	4.65	112.00
29	SER	172.31	58.61	68.23	8.33	5.57	111.96
30	THR	173.64	60.77	72.34	8.42	5.32	116.15
31	ASN	173.22	55.32	43.28	9.17	5.54	129.71
	ASN′	174.30	53.97	36.07	0.00	5.49	0.00
32	VAL	174.96	60.52	32.64	9.21	4.97	120.11
	VAL′	174.10	59.61	35.09	8.38	5.09	121.71
33	GLY	171.04	44.48	0.00	8.65	3.63	114.75
34	SER	173.28	58.15	67.50	8.22	5.23	114.36
35	ASN	174.14	52.78	41.17	8.78	5.84	122.16
36	THR	171.61	62.11	70.97	8.72	4.78	120.85
37	TYR	175.13	55.59	40.16	9.13	5.23	126.21

Table S3B: Side-chain chemical shifts in pH 5.3 lipidic fibrils.

1	LYS	Cγ 24.03, Cδ 28.31, Cε 43.28, Hγ2 0.84, Hδ2 1.12, Hε2 1.56
3	ASN	Cγ 179.52

4	THR	Cγ2 24.11
6	THR	Cγ2 22.96
9	THR	Cγ2 19.56, Hγ1 0.80
10	GLN	Cγ 34.49, Cδ 178.71
12	LEU	Cγ 27.82, Cδ1 25.63, Hγ 1.85
16	LEU	Cγ 28.02, Hγ 0.98
17	VAL	Cγ1 22.19, Cγ2 21.06, Hγ1 0.87, Hγ2 0.86
17	VAL′	Cγ1/2 21.10
18	HIS	Cδ2 118.54, Cε1 135.75
26	ILE	Cγ1 27.80, Cγ2 18.41, Cδ1 13.47, Hγ 0.50, Hδ1 0.62, Hγ12 1.51, Hγ13 0.87
26	ILE	Cγ1 27.33, Cγ2 16.44,
27	LEU	Cγ 29.41
30	THR	Cγ2 21.25, Hγ1 0.92
31	ASN	Cγ 178.16
32	VAL	Cγ1 20.84, Cγ2 21.65
32	VAL′	Cγ1 21.57, Cγ2 21.03
36	THR	Cγ 21.864, Hγ1 1.11
37	TYR	Cγ 129.68

Table S4: Summary of hIAPP polymorphs observed for wild-type and S20G mutant by cryo-EM. Free energy stability values were computed from PDB files, with images generated using scripts from Michael Sawaya, as used in the Amyloid Atlas webserver.[2] Peptide source (synthetic or recombinantly expressed), fibril growth conditions, and fold type are indicated below each polymorph. ΔG per fibril layer represents the total folding free energy change for a single middle layer, while ΔG per residue is the layer value divided by the number of ordered residues.

<table>
<tr>
<td>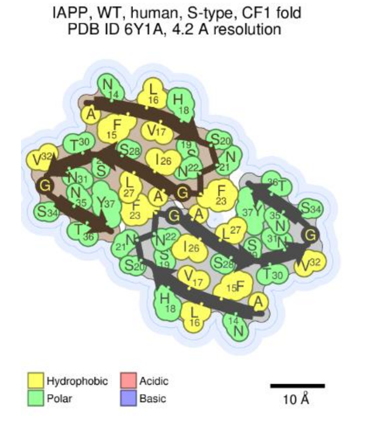
</td>
<td>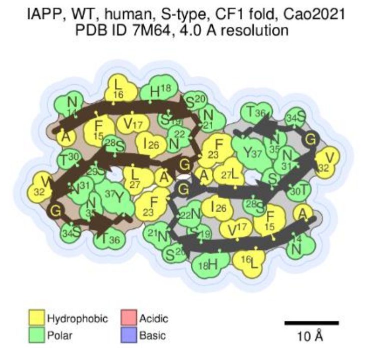
</td>
<td>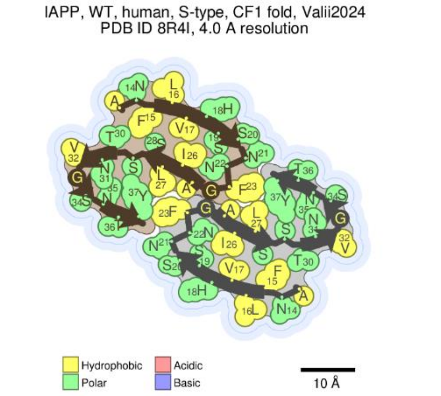
</td>
</tr>
<tr>
<td>

hIAPP: WT, synthetic
pH: 6.0
FOLD: CF1
TYPE: Homotypic
ΔG0 (kcal/mol)
-25.0 per layer, -0.50 per residue

</td>
<td>

hIAPP: WT, synthetic, seeded
pH: 7.4
FOLD: CF1
TYPE: Homotypic
ΔG0 (kcal/mol)
-27.0 per layer, -0.54 per residue

</td>
<td>

hIAPP: WT, synthetic
pH: 7.4
FOLD: CF1
TYPE: Homotypic
ΔG0 (kcal/mol)
-21.9 per layer, -0.44 per residue

</td>
</tr>
<tr>
<td>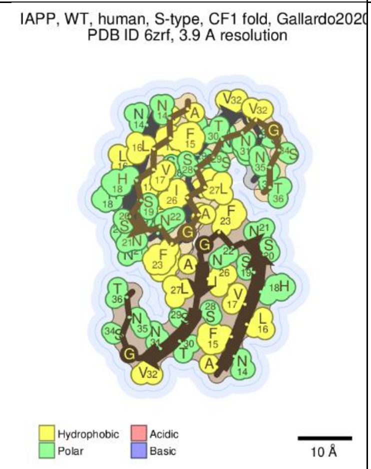
</td>
<td>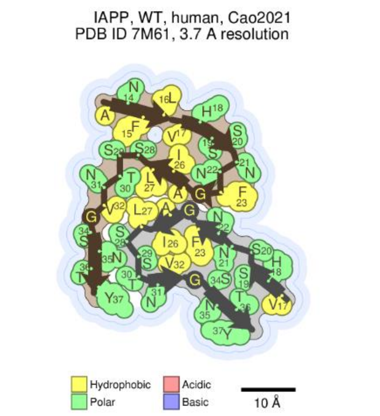
</td>
<td>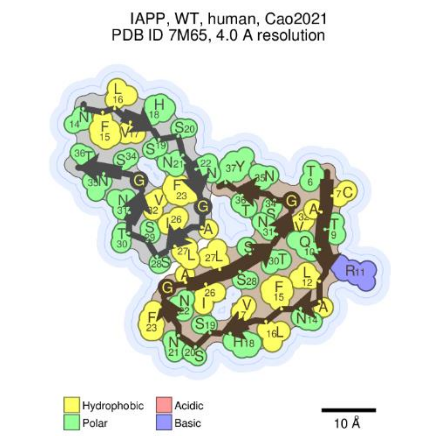
</td>
</tr>
<tr>
<td>

hIAPP: WT, synthetic
pH: 6.8
FOLD: CF1
TYPE: Homotypic
ΔG0 (kcal/mol)
-23.9 per layer, -0.33 per residue

</td>
<td>

hIAPP: WT, synthetic, seeded
pH: 7.4
FOLD: CF1, CF2
TYPE: Heterotypic
ΔG0 (kcal/mol)
-24.7 per layer, -0.54 per residue

</td>
<td>

hIAPP: WT, synthetic, seeded
pH: 7.4
FOLD: CF1, CF2
TYPE: Heterotypic
ΔG0 (kcal/mol)
-26.5 per layer, -0.48 per residue

</td>
</tr>
</table>

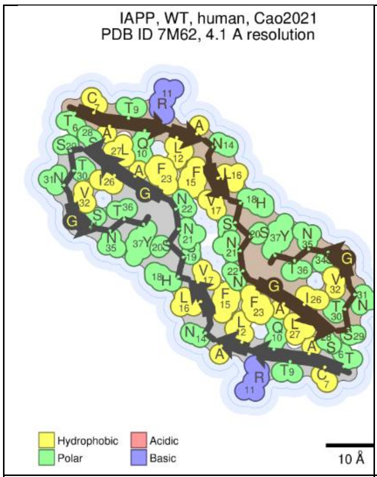

- **hIAPP:** WT, synthetic, seeded
- **pH:** 7.4
- **FOLD:** CF3
- **TYPE:** Homotypic
- **ΔG_0 (kcal/mol)**

-31.8 per layer, -0.50 per residue

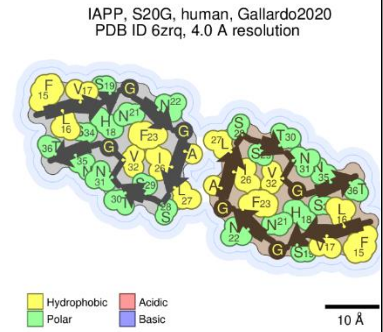

- **hIAPP: S20G**, synthetic
- **pH:** 6.8
- **FOLD:** CF2
- **TYPE:** Homotypic
- **ΔG_0 (kcal/mol)**

-22.4 per layer, -0.51 per residue

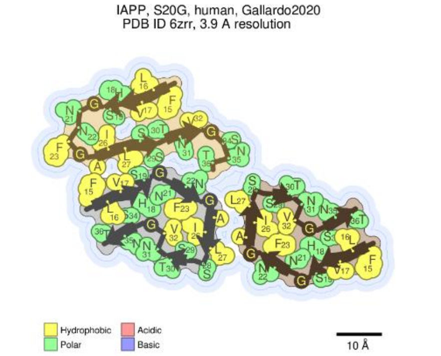

- **hIAPP: S20G**, synthetic
- **pH:** 6.8
- **FOLD:** CF1, CF2
- **TYPE:** Heterotypic
- **ΔG_0 (kcal/mol)**

-32.0 per layer, -0.48 per residue

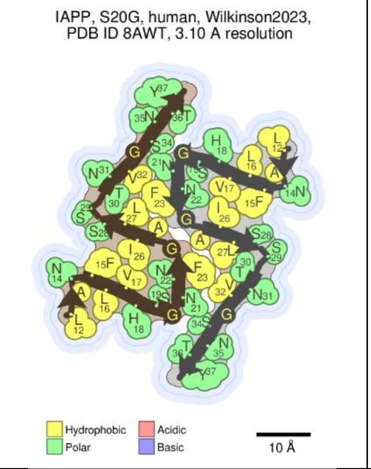

- **hIAPP:** S20G, synthetic
- **pH:** 6.8
- **FOLD:** CF1
- **TYPE:** Homotypic
- **ΔG_0 (kcal/mol)**

-31.8 per layer, -0.61 per residue

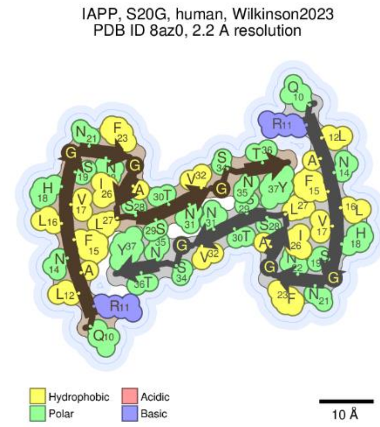

- **hIAPP:** S20G, synthetic
- **pH**: 6.8
- **FOLD:** CF1
- **TYPE:** Homotypic
- **ΔG_0 (kcal/mol)**

-30.3 per layer, -0.54 per residue

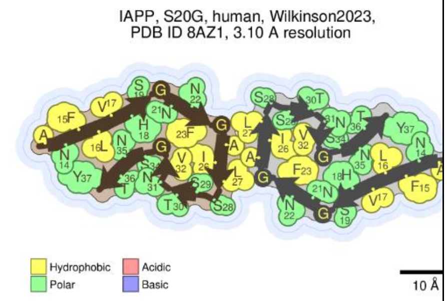

- **hIAPP:** S20G, synthetic
- **pH:** 6.8
- **FOLD:** CF2
- **TYPE:** Homotypic
- **ΔG_0 (kcal/mol)**

-28.6 per layer, -0.57 per residue

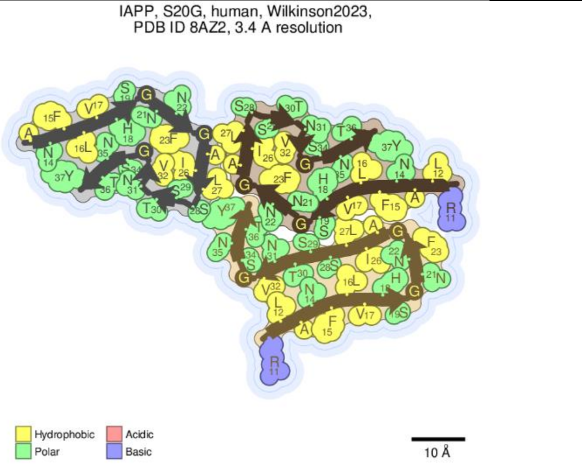

- **hIAPP:** S20G, synthetic
- **pH:** 6.8
- **FOLD:** CF1, CF2
- **TYPE:** Heterotypic
- **ΔG_0 (kcal/mol)**
  -45.6 per layer, -0.58 per residue

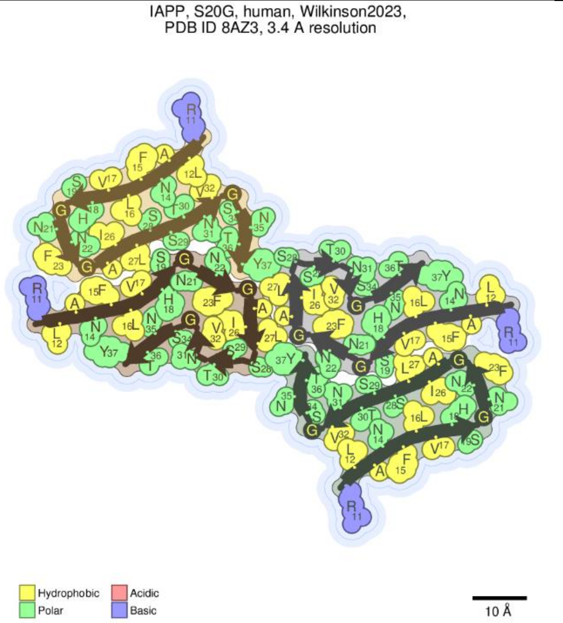

- **hIAPP:** S20G, synthetic
- **pH:** 6.8
- **FOLD:** CF1, CF2
- **TYPE:** Heterotypic
- **ΔG_0 (kcal/mol)**
  -61.0 per layer, -0.56 per residue

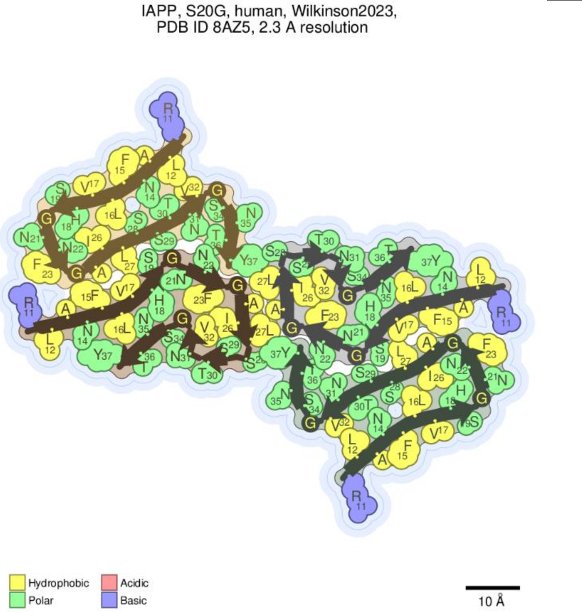

- **hIAPP:** S20G, synthetic
- **pH:** 6.8
- **FOLD:** CF1, CF2
- **TYPE:** Heterotypic
- **ΔG_0 (kcal/mol)**
  -57.8 per layer, -0.54 per residue

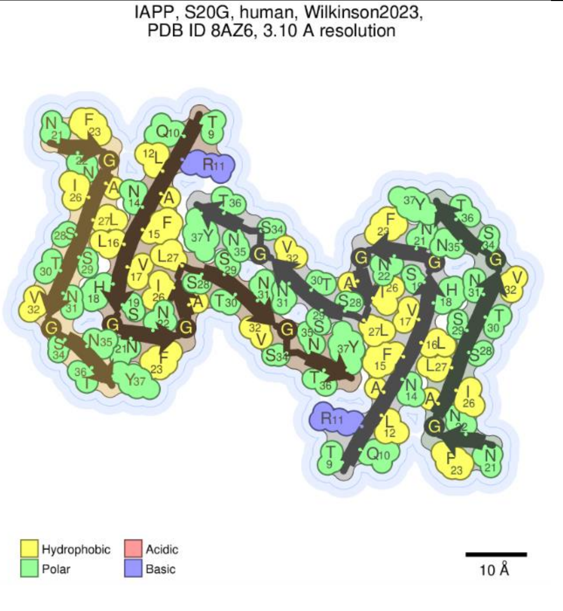

- **hIAPP:** S20G, synthetic
- **pH:** 6.8
- **FOLD:** CF1
- **TYPE:** Heterotypic
- **ΔG_0 (kcal/mol)**
  -45.3 per layer, -0.49 per residue

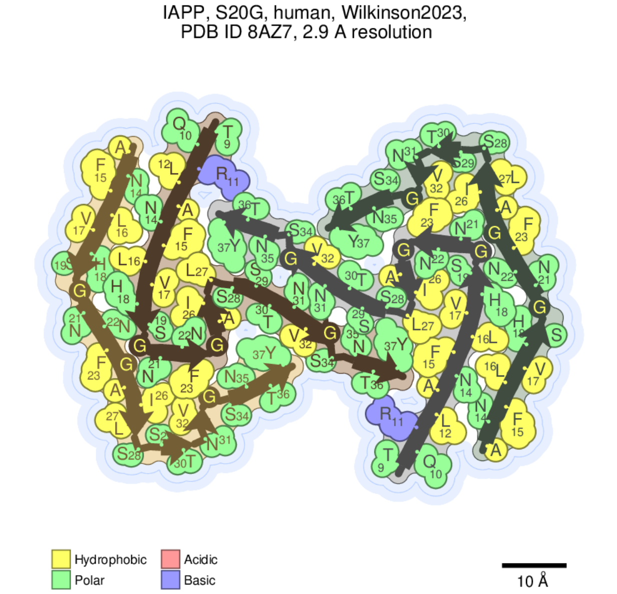	
• **hIAPP:** S20G, synthetic<br>• **pH:** 6.8<br>• **FOLD:** CF1<br>• **TYPE:** Heterotypic<br>• **ΔG$_0$ (kcal/mol)**<br>-65.7 per layer, -0.61 per residue	

REFERENCES

(1) Rodriguez Camargo, D. C.; Tripsianes, K.; Kapp, T. G.; Mendes, J.; Schubert, J.; Cordes, B.; Reif, B. Cloning, Expression and Purification of the Human Islet Amyloid Polypeptide (HIAPP) from Escherichia Coli. *Protein Expr. Purif.* **2015**, *106*, 49–56. https://doi.org/10.1016/j.pep.2014.10.012.

(2) Sawaya, M. R.; Sambashivan, S.; Nelson, R.; Ivanova, M. I.; Sievers, S. A.; Apostol, M. I.; Thompson, M. J.; Balbirnie, M.; Wiltzius, J. J. W.; McFarlane, H. T.; Madsen, A. Ø.; Riekel, C.; Eisenberg, D. Atomic Structures of Amyloid Cross-Beta Spines Reveal Varied Steric Zippers. *Nature* **2007**, *447* (7143), 453–457. https://doi.org/10.1038/nature05695.
